# Transfer and Reinforcement Learning as Support Paradigms for Human Activity Recognition in Indoor Environments: A Comprehensive Analysis of Trends, Impact and Future Directions

**DOI:** 10.3390/s26123751

**Published:** 2026-06-12

**Authors:** Paola Patricia Ariza-Colpas, Marlon-Alberto Piñeres-Melo, Ana Isabel Oviedo-Carrascal, David Díaz Jiménez

**Affiliations:** 1 Department of Computer Science and Electronics, Universidad de la Costa CUC, Barranquilla 080002, Colombia; 2 Faculty of Engineering in Information and Communication Technologies, Universidad Pontificia Bolivariana, Medellín 050031, Colombia; ana.oviedo@upb.edu.co; 3 Department of Systems Engineering, Universidad del Norte, Barranquilla 081001, Colombia; pineresm@uninorte.edu.co; 4 Department of Computer Science, University of Jaén, Campus Las Lagunillas, 23071 Jaén, Spain; ddjimene@ujaen.es

**Keywords:** transfer learning, reinforcement learning, human activity recognition, scientometric analysis, data analysis, activity datasets, smart environments

## Abstract

Human activity recognition—HAR—plays a crucial role in the lives of patients battling neurodegenerative diseases. These debilitating conditions, such as Alzheimer’s or Parkinson’s, affect individuals’ ability to perform daily tasks autonomously and safely. HAR technology offers an invaluable solution by enabling real-time monitoring and assistance, helping to maintain independence and quality of life for patients. Additionally, this technology provides a valuable data source for doctors and caregivers, allowing for more precise and personalized care, which can make a difference in managing and treating these neurodegenerative diseases. The objective of this review is to identify the contribution of Transfer Learning and Reinforcement Learning in supporting the processes of daily activity recognition, thus enhancing the quality of life for patients. As this is a trending topic, the literature surrounding it is quite dispersed, which is why this review aims to present the current line of research in this field. To carry out this analysis, the science tree paradigm was used, which establishes two fundamental stages of analysis. The first is delimited by scientometrics, where the leading countries in the application of such technologies can be identified. This review highlights the evolution in the use of transfer learning and reinforcement learning in HAR in the healthcare field, where these techniques have significantly improved the accuracy and adaptability of real-time monitoring systems. The studies reviewed indicate that transfer learning has allowed models to adapt to data variations without requiring large volumes of manual labeling, which is essential in clinical and patient monitoring contexts. Additionally, reinforcement learning has optimized decision-making in complex scenarios, enabling activity recognition systems to dynamically adjust monitoring parameters, enhancing detection and response to critical or unusual activities in multi-user environments. These advances demonstrate that, by integrating these approaches, greater personalization and robustness can be achieved in human activity recognition, thereby improving the quality of life for patients in clinical settings.

## 1. Introduction

HAR has emerged as a crucial field of study in today’s context, marked by rapid digitalization and the increasing integration of intelligent technologies across various sectors [1]. This discipline focuses on identifying and analyzing human actions and behaviors through data captured from sensors, video streams, and other sources, thereby enabling a deeper understanding of human needs and enhancing the performance of systems and services that interact with people [2]. HAR has found wide-ranging applications in domains such as healthcare, entertainment, security, medical assistance, and industrial automation [3]. Advancements in technologies like artificial intelligence and machine learning have significantly expanded the capabilities of HAR, particularly using computer vision, deep learning, transfer learning, and reinforcement learning [4,5]. These techniques have enabled more accurate, efficient, and adaptive recognition of human activities, even in complex or unstructured environments.

The COVID-19 pandemic further underscored the importance of HAR by highlighting the critical need to understand how individuals carry out their daily routines to ensure safety, well-being, and public health [6]. In this context, automatic activity recognition became essential in applications such as patient monitoring, social distancing, and remote assistance—thereby amplifying scientific interest in the field. Despite the growing volume of research on HAR, there remains a need for a systematic perspective to identify key trends, prominent authors, collaborative networks, and emerging thematic lines that have shaped the development of this domain. To address this gap, the present study undertakes a comprehensive scientometric analysis based on the Tree of Science (ToS) metaphor. This framework enables the classification of foundational works as “roots,” consolidated contributions as the “trunk,” and emerging lines of research as the “leaves.”

The main objective of this work is to analyze the key trends in scientific research on HAR, with a particular focus on the role of transfer learning and reinforcement learning techniques. To achieve this aim, the study first identified the most relevant HAR-related publications indexed in the Web of Science (WoS) and Scopus databases between 2010 and 2025. It then characterized the scientific output by analyzing the most prolific and influential authors, countries, and institutions. Citation and co-authorship networks were also examined using scientometric tools to map collaborative dynamics within the field. Finally, the Tree of Science methodology was applied to classify the collected works into seminal, consolidated, and emerging contributions, offering a structured overview of the thematic and methodological evolution of HAR research. The study was conducted by applying targeted search equations in the WoS and Scopus databases, followed by duplicate removal and consolidation into a unified dataset. The analysis was performed using the R package tosr, version 4.3.1 (Beagle Scouts), released on 16 June 2023 [7], which operationalizes the Tree of Science framework. This approach allowed for a reconstruction of the field’s intellectual development, its most impactful contributions, and its current societal relevance.

The review is organized into three main sections. The first describes the methods and procedures used in the scientometric analysis. The second presents the results, including metrics such as country-level productivity, most cited authors, and knowledge networks. The final section interprets the development of the field through the ToS framework, distinguishing between roots, trunk, and leaves to offer a comprehensive understanding of the thematic and methodological evolution of human activity recognition research.

## 2. Theoretical Information

### 2.1. Overview of HAR

HAR is an emerging field in science and engineering that aims to identify, classify, and in some cases, predict actions performed by individuals based on data collected from various sensors [7]. HAR has gained significant relevance due to its broad range of applications in areas such as healthcare, security, elder care, home automation, sports, and, more recently, human–robot interaction. In this context, recognizing activities in indoor environments presents a unique set of challenges and opportunities, particularly in terms of sensor selection, data acquisition, and the implementation of machine learning models for analysis.

#### 2.1.1. Types of Activities in HAR

HAR in indoor environments involves a thorough classification of the activities people perform in these spaces. Generally, activities can be grouped into the following categories—see Table 1.

#### 2.1.2. Sensors Used in HAR for Indoor Environments

Success in recognizing human activities in indoor environments largely depends on the sensors used to capture relevant data. The following are the main types of sensors employed in HAR, along with their characteristics and specific applications in indoor settings—see Table 2.

#### 2.1.3. Datasets for HAR in Indoor Environments

The development and validation of HAR algorithms require high-quality datasets that accurately represent human activities in real-world settings. In the context of indoor environments, several datasets are widely used by the scientific community. The following are some of the most widely referenced datasets, see Table 3:

### 2.2. Transfer Learning in HAR

Transfer learning is a machine learning paradigm that facilitates knowledge reuse from a source domain or task to improve performance in a different but related target domain or task. In the field of HAR, transfer learning has gained increasing relevance due to the high cost and complexity of collecting large-scale, labeled datasets across varying contexts, such as different environments, sensor placements, or user populations [41,42]. Traditional HAR models often suffer from poor generalization when deployed in conditions different from those in which they were trained, such as changes in sensor modality, location, or demographic characteristics of the users. Transfer learning mitigates this limitation by enabling models to adapt learned representations, parameters, or instances from a source dataset to a new target dataset, often with limited labeled data available for the latter [43,44]. In HAR, transfer learning approaches are commonly categorized based on the type of knowledge that is transferred:

- Feature-based transfer learning aims to extract domain-invariant features that remain consistent across source and target domains. Techniques such as Domain-Adversarial Neural Networks (DANN) [45] and Maximum Mean Discrepancy (MMD)-based adaptations [46] are widely used to reduce distributional divergence between domains.
- Instance-based transfer learning focuses on selecting or reweighting source instances that are most relevant to the target domain. Algorithms like TrAdaBoost [47] and Kernel Mean Matching (KMM) help reweight source samples to align them with the target distribution.
- Parameter-based transfer learning involves fine-tuning pretrained models—often deep neural networks—on the target domain. Pretrained Convolutional Neural Networks (CNNs) and Recurrent Neural Networks (RNNs) trained on large HAR datasets can be adapted with minimal labeled data using techniques like layer freezing and learning rate scheduling [48].
- Relational transfer learning transfers inter-feature or inter-activity relationships from one domain to another. This is especially relevant in multi-activity recognition, where the temporal or semantic relationships among activities (e.g., sitting often follows walking) can be shared across datasets [49].

These methods have been applied in scenarios such as cross-user HAR, cross-sensor adaptation, and domain generalization across indoor/outdoor or lab/real-world environments, see Table 4.

### 2.3. Reinforcement Learning in HAR

Reinforcement Learning (RL) is a branch of machine learning in which an agent learns to make decisions by interacting with its environment and receiving feedback in the form of rewards or penalties [50]. In the context of HAR, RL techniques are employed to enhance activity recognition systems by optimizing their adaptability to changing conditions, users, and environments. RL is particularly effective in scenarios where data is limited or where the system must adapt in real time to new activities or contexts (see Table 5).

## 3. Methods

To carry out this analysis, a bibliometric review methodology was employed to identify and highlight the most significant scientific contributions in the field of HAR, particularly in its applications to the healthcare sector. The Web of Science (WoS) and Scopus databases were selected due to their extensive collections of peer-reviewed academic publications. The datasets retrieved from both sources were merged using specialized tools such as Bibliometrix [56] and the Tosr R package, enabling an integrated and consensus-based view of the scientific output in this domain. It is important to note that the COVID-19 pandemic was not included as a search term in the bibliometric query. However, a marked increase in research interest and scientific output related to HAR in healthcare contexts was observed during the pandemic period. This reflects the growing need for non-intrusive, remote monitoring technologies that can support patient care, especially for vulnerable populations, in scenarios where physical interaction is limited. By consolidating records from both databases, this study offers a comprehensive overview of the current research landscape and emphasizes the most impactful contributions of HAR in healthcare—particularly those that gained relevance in response to the challenges posed by the global health crisis and the transition toward digitally assisted care models.

**Figure 1:**
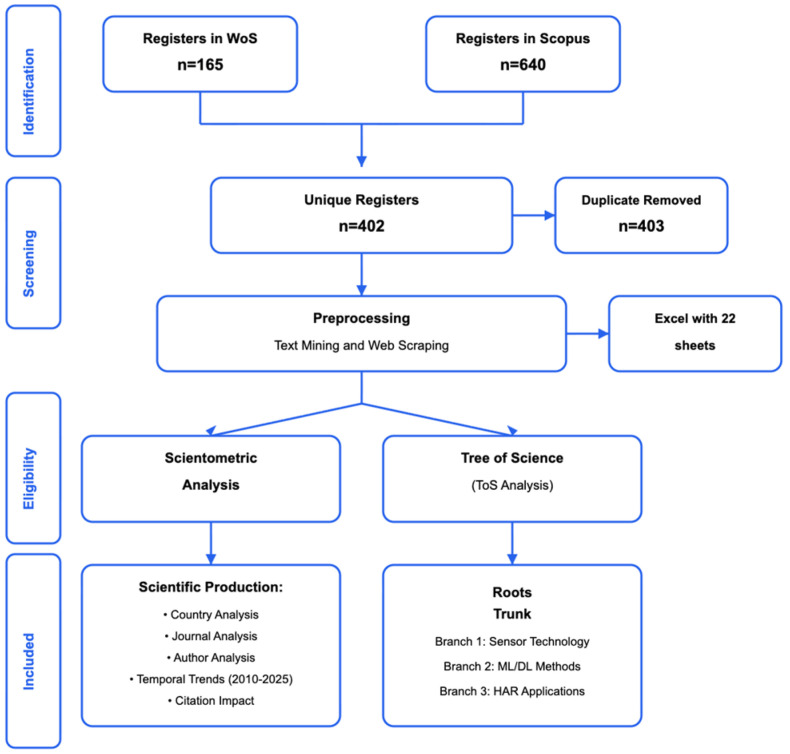
PRISMA diagram for preprocessing data.

The bibliometric review was based on a combined Boolean query that incorporated two sets of keywords using the AND operator. The first set included “Transfer Learning” OR “Reinforcement Learning”, while the second set comprised “HAR” OR “Human Activity Recognition” OR “ADL” OR “Activity Daily Living”. The search was restricted to the title, abstract, and keyword fields in both the Scopus and Web of Science (WoS) databases, and covered the period from 2010 to the present, to capture the most recent advances in the application of machine learning techniques for human activity recognition. Initially, this search retrieved 640 documents from Scopus and 165 from WoS, yielding a preliminary combined total of 805 records before filtering and duplicate removal. After a thorough data integration and cleaning process using bibliometric tools such as Bibliometrix (R package): 4.2.3 and VOS viewer package 1.6.20, a final consolidated set of 402 unique publications was obtained after removing 403 duplicates. These filtered records were then analyzed and included in this study following the PRISMA methodology for systematic reviews, see Figure 1. The completed PRISMA 2020 checklist is provided as Supplementary Material, Table S1.

The 405 documents are distributed across several categories as follows: Journal Articles (175, 43.21%), Conference Papers (160, 39.51%), Conference Reviews (56, 13.83%), Review Articles (7, 1.73%), Early Access Papers (2, 0.49%), Book Chapters (3, 0.74%), and Short Surveys (2, 0.49%). This distribution shows that most publications continue to appear as Journal Articles and Conference Papers, reflecting the central channels through which research in this field is commonly disseminated. During the screening process for literature on Human Activity Recognition (HAR), documents that did not present original research or empirical data were excluded to ensure that the review remained grounded in solid scientific evidence. Non-English publications were removed to guarantee clarity and accessibility for analysis. Additionally, studies that did not address topics directly related to HAR were excluded to maintain thematic relevance and preserve the focus of the review. These exclusion criteria were applied to produce a precise and targeted overview of the scientific landscape concerning HAR in the healthcare sector.

To conduct the scientometric analysis presented in this article, the process was structured into two distinct stages. In the first stage, a panoramic overview of the various applications developed within the field was provided. This included an analysis of scientific output by country, journal, and author, offering a global perspective on research activity in this domain. In the second stage, specific scholarly contributions were examined using the Tree of Science (ToS) metaphor, which classifies publications as roots, trunk, or leaves according to their conceptual influence and position in the scientific discourse. In this article, the overall methodological structure followed a flow like the PRISMA model to document all phases of information consolidation. Data analysis was conducted using R Studio version 2023.12.1, supported by a custom script developed by Core of Science. This tool facilitated the extraction and processing of key bibliometric indicators from the records obtained through the search strategy. Together, these two stages enabled a comprehensive and systematic overview of scientific production in the targeted area of study.

### 3.1. Scientometric Analysis

Scientometrics is employed to perform quantitative analysis of scientific output, allowing the identification of key elements such as author collaboration networks, citation structures, and annual publication trends within a given research context. The primary methods used in this study include citation analysis [57], collaboration network analysis [58], and intellectual structure analysis [59]. The objective of this analysis is to evaluate the scientific contributions to a specific area of knowledge by examining publication trends by country, annual research output, and the journals most frequently publishing on the topic. The analysis is based on consolidated sources such as Scopus and Web of Science (WoS), and incorporates clustering techniques to enable a more descriptive and comprehensive understanding of the research landscape.

Although the review presented in this study was based on a rigorous and systematic search method designed to capture the most significant contributions regarding transfer learning and human activity recognition in intelligent environments, it is important to acknowledge certain inherent limitations of the process. In particular, the use of specific combinations of keywords and controlled terms, while useful for establishing thematic boundaries, may have excluded significant research whose terminology did not directly match the descriptors used. In interdisciplinary fields, such as artificial intelligence applied to healthcare, this phenomenon is especially common. In these fields, similar ideas can be referred to using different expressions; for example, “motion pattern recognition” instead of “human activity recognition,” or “knowledge recycling” instead of “transfer learning.” Additionally, the reliance on indexed bibliographic sources, especially databases such as Web of Science and Scopus, may have limited the scope of emerging technical literature, including technical reports with regional impact, conference proceedings with restricted access, or preprints. Although measures were implemented to mitigate these risks, such as including synonyms and cross-checking highly cited references, it is acknowledged that some works may have been omitted due to their nomenclature or dissemination channel, which could not be filtered. In future studies, it is recommended to combine this analysis with semantic mining techniques and ontological expansion to achieve a broader range of terms and ensure more comprehensive coverage of the state of the art.

### 3.2. Tree of Science

This review adopts the Tree of Science (ToS) metaphor, which offers a symbolic framework for organizing scientific literature. In this model, articles positioned at the roots represent the theoretical and foundational underpinnings of a research topic. The trunk contains works that have contributed to the development and consolidation of essential concepts, while the leaves reflect current application trends and emerging directions in the field, illustrating how technologies supporting human activity recognition are evolving. This methodology has been widely applied in various disciplines, including economics [60], education [61], and marketing [62], among others, due to its ability to visualize the evolution and structure of scientific knowledge.

## 4. Impact and Development Through Time

**Figure 2:**
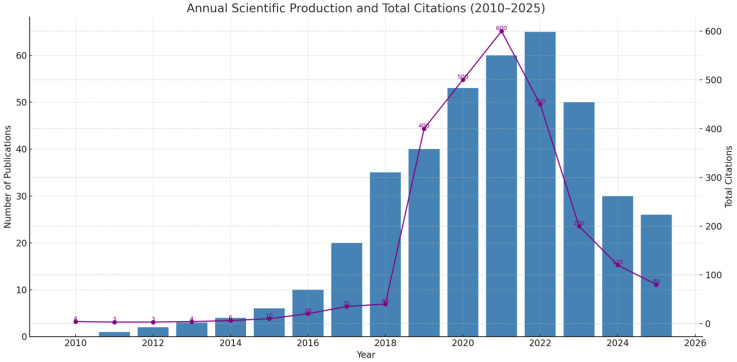
Total Scientific Production vs. Total Citations.

Figure 2 illustrates the evolution of scientific production related to Human Activity Recognition (HAR) over the past 15 years. As evidenced by the trend, there has been a steady increase in the number of publications, particularly in recent years. The earliest contributions, published around 2010, received minimal citations, indicating limited early adoption of the topic. However, beginning in 2016, a noticeable rise in research output is observed across both the Web of Science (WoS) and Scopus databases, reflecting growing academic interest and the consolidation of HAR as a research field.

From the figure, we can identify that the research topic addressed in this project has evolved through three distinct phases of development, which will be described in the following section. The authors highlighted in this analysis are those whose work has demonstrated a strong reception within the academic community. This is reflected through high citation counts, recurring influence in collaborative networks, or consistent contributions across different stages of the field’s evolution. Their publications have played a foundational, consolidating, or emergent role in shaping the scientific discourse around HAR.

Initial Phase (2010–2016): During this early stage, scientific output and citation impact remained relatively modest, as is common during the formative years of an emerging research domain. The gradual increase in publications reflects the progressive consolidation of HAR as a relevant area of inquiry, particularly within healthcare applications. Research from this period laid the groundwork for applying intelligent systems to assist with Activities of Daily Living (ADLs), with a strong emphasis on enhancing the accuracy of behavioral recognition in individuals with neurodegenerative or cognitive impairments. One of the central challenges addressed during this phase was the distinction between anomalous behaviors and typical variations in daily routines—an issue critical for the reliability of assistive technologies. Proposed methodologies in this stage often incorporated advanced tools such as the Internet of Things (IoT) and data analytics, aiming to improve the precision of error detection mechanisms in the execution of ADLs. These early innovations not only provided a more refined approach to activity monitoring but also demonstrated potential for integration into smart home and remote care systems, contributing to improved quality of life and better support for caregivers. Several foundational contributions from this phase are summarized in Table 6.

Development Phase (2017–2020): This phase was characterized by accelerated growth in both scientific output and research impact, marking a shift from exploratory efforts to the consolidation of HAR as a structured and applied research field. Significant advancements were made through the integration of Internet of Things (IoT) and Artificial Intelligence (AI), particularly in healthcare contexts such as the monitoring and management of neurodegenerative diseases. The use of deep learning models became increasingly effective in processing heterogeneous sensor data, enabling more accurate recognition and classification of daily human activities. This enhanced capability supported not only personalized healthcare services and timely interventions but also improved patient autonomy and treatment outcomes. Additionally, the ability to generate more reliable behavioral insights contributed to better clinical decision-making and more precise tracking of disease progression, see Table 7.

Strengthening Phase (2021–Present): Since 2021, the field of HAR has entered a phase of consolidation. Although the pace of publications and citations has declined compared to the 2020 peak, there is a clearer focus on research quality and the maturity of proposed approaches. This phase is marked by the integration of advanced machine learning techniques, particularly multi-sensor data fusion, to address challenges such as noise, subject variability, and adaptation to dynamic contexts.

There is also a growing emphasis on developing more robust, scalable, and efficient systems for real-world applications—especially in healthcare, intelligent assistance, and rehabilitation—highlighting the need for models capable of learning with minimal or no supervision, see Table 8.

## 5. Results

### 5.1. Pioneering Countries in the Development of the Research Line of Human Activity Recognition

The analysis of leading countries in human activity recognition research allows for the identification of global Centers of Excellence in advanced HAR technologies—most notably China and the United States, which distinguish themselves through high research output and impact, respectively. Additionally, these findings enable the evaluation of the effectiveness of national policies and strategies aimed at promoting research and technological development, illustrating how targeted policy interventions can influence the quality and relevance of scientific production. Finally, the results aid the establishment of benchmarking standards in the HAR domain, offering a comparative framework that other countries may adopt to assess their own progress and performance in HAR research. This analysis not only provides a comprehensive global overview of the current state of HAR but also lays the groundwork for future international collaborations and the advancement of unified global standards in the development and deployment of human activity recognition and monitoring technologies, see Table 9.

Table 9 provides a comparison of research productivity and impact across various countries, measured by the number of scientific publications, citations received, and their distribution across journal quartiles (Q1 to Q4), which indicate the quality or prestige of the journals where these articles were published. Analyzing the table, China leads in terms of publication production with 51 publications. However, in terms of citation impact, China ranks second with 17.65%, suggesting considerable but not the highest citation influence. This observation suggests that China has a high production volume, but each publication, on average, has less impact than some of its counterparts. The article by Wang [4], published in 2017 in Information Fusion, introduces an innovative kernel fusion technique based on Extreme Learning Machine (ELM) for activity recognition across different locations, addressing a key challenge in human activity recognition: contextual variability between environments. The fundamental contribution lies in the kernel fusion approach, allowing effective integration of sensor data from multiple sources. The fast-learning capabilities of ELM neural networks enhance efficiency and handle large data volumes, improving activity recognition performance, especially in scenarios with significant differences in spatial arrangements and sensory environments. This technique is applicable in health monitoring, elderly care, home security systems, and IoT applications where human activity recognition is critical.

The United States, in contrast, shows lower production with 41 articles compared to China but significantly leads in citations at 23.78%. This high citation rate indicates that, despite fewer publications, the impact per article is considerably higher. Additionally, their publications are more frequently found in higher-quartile journals (Q1 and Q2), reflecting higher quality and prestige. The 2018 article by Ramasamy, Ramamurthy, and Roy [2], published in Wiley Interdisciplinary Reviews: Data Mining and Knowledge Discovery, stands out as a key reference, offering a detailed analysis of recent advances and trends in machine learning applied to HAR. This comprehensive review article serves as a critical synthesis of research methodologies and technological innovations that have emerged to improve accuracy and efficiency in identifying human activities through sensor data and machine learning. The authors underscore how integrating sophisticated algorithms with the growing availability of sensor data has significantly advanced HAR. Various approaches, including supervised and unsupervised learning models, are discussed, and their application in real-world scenarios is explored. Furthermore, the authors address current challenges in data collection and processing, as well as ethical and privacy concerns associated with capturing human activity data. They also critically assess the utility of publicly accessible HAR datasets, emphasizing the importance of robust and diverse databases for training and validating machine learning models. Additionally, the future implications of HAR research are considered, including the potential for personalized healthcare and the adaptation of smart environments to individual needs.

India ranks third with 29 publications, but its citation rate is relatively low (4.32%), a factor that may suggest that, despite a good amount of output, its international impact might be more limited. The distribution of its publications across quartiles also indicates that a significant portion is in lower quartiles (Q3 and Q4). The article by Khan, Roy, and Misra [5], presented at the IEEE PerCom conference in 2018, represents one of the most notable contributions from India. It addresses a crucial challenge in HAR: domain adaptation in scalable HAR using deep learning techniques. Domain adaptation involves the process of applying a machine learning model trained in one domain (an environment with specific characteristics and data) to another domain where conditions may vary significantly. In their study, the authors propose a deep learning-based solution that allows HAR models trained on one dataset to adapt and perform effectively on other datasets without requiring intensive manual labeling. This is particularly relevant when considering the different environments, sensors, and conditions under which human activity data can be collected. The main innovation of their work lies in the use of deep learning techniques to mitigate the domain mismatch problem, which occurs when training and testing data come from different distributions. The authors’ domain adaptation approach allows the model to generalize better and be effectively applied to new environments, which is crucial for real-world applications where collecting and labeling large volumes of sensor data is impractical and costly.

The United Kingdom and South Korea have fewer publications (17 and 16, respectively) but maintain a relatively healthy citation profile (3.84% and 4.49%, respectively), indicating that their research is well-received within the global scientific community. The distribution of publications in higher quartiles suggests notable quality and relevance in their research work. One of the significant contributions from the UK comes from the study by Shah and Fioranelli [78], presented at the 2019 International Radar Conference. Their study explores the portability of datasets in human activity recognition using Frequency Modulated Continuous Wave (FMCW) radar. The research represents an advance in activity recognition through radar technology, which offers a non-invasive alternative that is less susceptible to privacy concerns compared to cameras or wearables. The paper focuses on evaluating how effectively a model trained on one FMCW radar dataset can be applied to another without significant loss of accuracy. This is crucial for developing human activity recognition systems that are effective across different environments and individuals without requiring extensive recalibration or data re-labeling. The research by Shah and Fioranelli demonstrates the feasibility of using FMCW radar to detect and classify common human activities, which is promising for applications in assisted living, security, and health, where continuous and accurate human activity recognition is essential. Additionally, the study’s preliminary results emphasize the importance of dataset portability in HAR research, an aspect that can accelerate the adoption of radar-based solutions in healthcare and home well-being monitoring. This approach can enhance early detection of abnormal activity patterns, which could be indicative of progressing neurodegenerative disease, and allow for timely adjustment of necessary treatments or interventions.

Japan and Germany follow with even fewer publications, but with a notably higher citation profile, especially Japan, which stands out with a citation rate of 10.37%. This indicates that, although their output is limited, the impact of their publications is significant. Both countries have a presence in Q1 and Q2 but not in Q3 and Q4, possibly reflecting a focus on high-quality publications. A notable contribution from these countries is the work of Li, Shirahama, Nisar, Huang, and Grzegorzek [79], who authored a 2020 article published in Sensors, addressing a crucial issue in time-series data processing generated by various sensors: the application of deep learning and transfer learning to improve sensor modality classification. In the context of an explosive increase in sensor data generation across various applications, from health monitoring to predictive maintenance in industry, this study presents an innovative approach that significantly enhances the efficiency and accuracy of data classification. The authors introduce a deep transfer learning model that, instead of requiring extensive retraining or data collection for each new sensor type or context, allows pre-trained models to adapt effectively to classify data from new sensor modalities. This methodology is particularly valuable given the diversity and volume of sensor-generated data, where variability between sensor types can pose a significant challenge to traditional analysis. The central innovation of the article lies in its ability to address the problem of a lack of labeled data for each specific sensor modality, a common challenge in machine learning. By using transfer learning, the study demonstrates how knowledge gained in one classification task (source domain) can be applied to a different but related task (target domain), thereby reducing the need for large labeled datasets and costly annotation processes.

The article by Hernandez et al. [80], published in 2020 in SN Computer Science, stands as one of Germany’s significant contributions. It provides a comprehensive literature review on the application of transfer learning in human activity recognition (HAR) using mobile devices and wearables, along with environmental technologies. This study systematizes current knowledge on improving activity detection and classification through transfer learning, a technique that allows machine learning models applied to one task to be reused for another, reducing the need for extensive task-specific training data. The article addresses a major challenge in HAR: data variability caused by different users, devices, device orientations, and environmental contexts. This variability makes it difficult to generalize models trained on one dataset to situations not seen during training. By applying transfer learning techniques, the authors aim to improve machine learning models’ ability to adapt to these variations, making activity recognition more robust and generalizable. Moreover, the article also discusses the importance of integrating environmental sensor data with mobile and wearable data to enrich the context of activity recognition, providing a more holistic view of the situations in which activities are performed. This multidimensional approach is crucial for applications where the environmental context can significantly influence the interpretation of activity data, such as in health and wellness monitoring in home and professional settings.

Canada, Iran, and Singapore present a similar number of publications but with notable differences in citation counts and quartile distribution. Canada has a low citation profile and no presence in Q1, suggesting a need to increase research impact. Iran shows a more uniform distribution across quartiles, indicating a broader quality spectrum. Singapore, with an impressive 7.79% citation rate and Q1 publications, demonstrates significant impact and quality in research. Among Canada’s most relevant authors are Nikpour and Armanfard [81], who presented their work at the 2021 IEEE International Conference on Systems, Man, and Cybernetics (SMC), introducing a novel methodology for skeleton-based activity recognition using deep reinforcement learning for joint selection. In the field of HAR, skeleton-based representation has gained popularity due to its ability to capture complex human motion dynamics with a relatively simple and efficient representation. However, one of the key challenges in this approach is determining which joints are most informative for recognizing specific activities, as not all joints contribute equally to the performance of an activity. Nikpour and Armanfard address this challenge by developing a system that applies deep reinforcement learning techniques to automatically identify the most relevant joints for activity recognition. Reinforcement learning, a branch of machine learning, enables an agent to learn to make optimal decisions through interaction with an environment—in this case, by selecting an optimal subset of joints that enhance recognition performance. The key innovation of their approach lies in the use of a reinforcement learning policy that guides joint selection, allowing the system to dynamically adjust which joints to consider based on each joint’s contribution to activity recognition accuracy. This selection process not only improves model efficiency by reducing the dimensionality of the input data but also increases accuracy by focusing on the most informative features of the skeleton.

**Figure 3:**
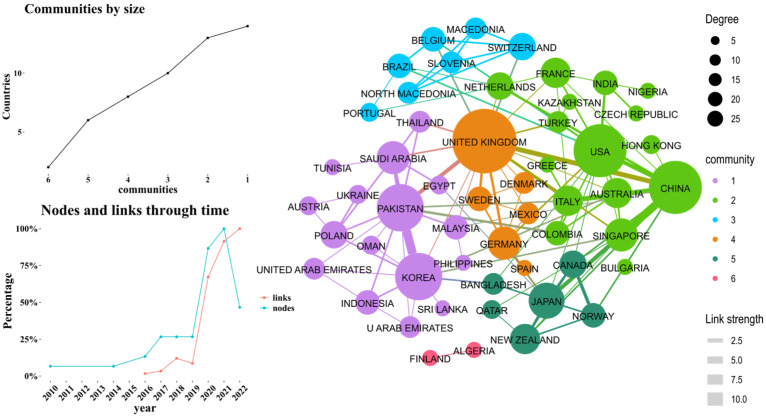
Communities of countries conducting research.

The article by Soleimani and Nazerfard [81], published in 2021 in Neurocomputing, is a key contribution from Iran to the development of this field, exploring the use of Generative Adversarial Networks (GANs) to address the challenge of cross-subject transfer learning in HAR systems. The study tackles one of the main difficulties in HAR: variability between subjects. Their central proposal involves using GANs to generate synthetic data that mimic the characteristics of unseen subjects during training, improving the generalization of HAR models across different individuals. The incorporation of GAN-generated data into HAR model training significantly enhances the model’s ability to recognize activities in previously unseen subjects, which is crucial for practical HAR applications where collecting and labeling large amounts of data for every new user is impractical. Finally, the article “Adarnn: Adaptive learning and forecasting of time series,” presented by Du, Wang, Feng, Pan, Qin, Xu, and Wang [82] at the 30th ACM International Conference on Information and Knowledge Management in October 2021, is a significant contribution from Singapore. It introduces a novel model called Adarnn (Adaptive Recurrent Neural Network) aimed at improving time series prediction accuracy through an adaptive approach that dynamically adjusts its structure in response to changes in data patterns. Adarnn improves both flexibility and robustness by addressing the challenges of non-stationarity and variability in real-world time series data, see Figure 3.

Figure 3 represents how various communities (countries) have interacted with each other over the years in terms of scientific collaborations. The first community, in purple, is led by Korea [49], with strong relations with Pakistan, and is distinguished by its sustained research in the development and application of advanced deep learning and transfer learning models for context-aware human activity recognition and intelligent environmental control systems. For instance, Gong et al. [50] proposed MetaSense, a few-shot adaptation method for deep mobile sensing, allowing HAR models to quickly adapt to new, untrained conditions with minimal data. This technique is crucial for dynamic environments such as smart homes and offices. Similarly, Jung et al. [83,84] introduced a deep learning-based real-time temperature control system centered on occupant preferences, which proactively adjusts indoor conditions based on learned behavioral patterns. These systems represent a major leap beyond traditional HVAC mechanisms by integrating human-centric sensing and prediction for energy-efficient comfort management. Moreover, the Korean research network has also contributed significantly to the advancement of spatiotemporal recognition frameworks for complex and overlapping activity classes, as demonstrated by Bilal et al. [85], and the use of semi-supervised active transfer learning to improve HAR performance with limited labeled data [86]. Feature representation transfer for HAR was explored by Mutegeki & Han [87], while Nguyen et al. [88] provided a comprehensive overview of transfer learning strategies for wireless networks, emphasizing cross-domain generalization—an essential capability for real-world deployment. Additional innovations include cross-domain recognition using autoencoders [89], sensor duty cycling optimization with reinforcement learning [90], and hybrid domain adaptation strategies to manage heterogeneous sensor setups [91]. Finally, the integration of mobile robotics and user-state-specific thermal control in real smart environments [92] points toward a future where intelligent agents autonomously learn and respond to occupants’ thermal comfort needs. These collective efforts illustrate Korea’s leadership in developing adaptive, energy-conscious, and user-centered technologies. Their work not only enhances individual comfort and energy efficiency but also aligns with broader global goals for sustainable urban development and climate change mitigation. The ability to dynamically adjust environmental conditions based on human activity and preferences signifies a fundamental shift toward personalized, intelligent living environments.

The second community, in light green, is led by China, with a significant number of collaborative publications with the United States, focusing on advancing transfer learning and deep learning techniques for HAR. This community addresses one of the central challenges in HAR: the heterogeneity of sensor data arising from variations in subjects, devices, and environmental contexts, which can severely limit model generalization. A key contribution comes from Ding et al. [93], who conducted an empirical study to evaluate and improve deep transfer learning models, proposing methods to increase their adaptability and robustness across diverse domains. Expanding on this, Fu et al. [94] explore personalized HAR using integrated wearable sensors and transfer learning, enabling models to fine-tune based on individual-specific data while still maintaining generalizability. Meanwhile More recently, Qin et al. [95] introduced an adaptive spatiotemporal transfer learning approach to improve cross-dataset recognition performance by dynamically adjusting to temporal and spatial discrepancies in sensor data. Shi et al. [96] advanced the application of deep learning in HAR using radar sensing technologies, broadening the modalities available for activity recognition. Wang et al. [97] contributed strategies for cross-domain knowledge transfer that reduce dependency on large labeled datasets, improving scalability across real-world applications. Another notable advancement is the integration of virtual and real wearable sensors, as proposed by Pei et al. [98] within the MARS framework, which employs multi-domain deep learning for more robust recognition in mixed-reality settings. Similarly, Zhang et al. [99] employed graph-based few-shot learning with channel state information (CSI) to address low-resource HAR scenarios, highlighting the potential of graph neural networks to improve learning efficiency. In addition, Lu et al. [100] proposed a novel substructural optimal transport method for cross-domain activity recognition, effectively aligning data distributions between source and target domains at a finer structural level. Chen et al. [101] further contributed to cross-domain knowledge transfer by reducing dependency on large labeled datasets and improving scalability across real-world applications. Together, these contributions underscore China’s leadership in developing adaptable, scalable, and data-efficient HAR models. The focus on cross-domain and cross-device generalization, combined with personalized and context-aware methods, enhances the applicability of HAR in critical areas such as remote patient monitoring, smart homes, and elder care. These innovations are essential for overcoming barriers related to data variability and annotation scarcity, thereby enabling broader deployment of HAR systems in real-world environments.

The third community, highlighted in light blue, is primarily represented by Belgium, Macedonia, and Switzerland. This group has made significant contributions to advancing HAR by emphasizing the practical application of deep learning techniques in real-world healthcare scenarios. One notable line of research focuses on the remote characterization of gait in patients with multiple sclerosis (MS) using smartphones, as demonstrated by Creagh et al. [102]. Their work not only illustrates the potential of mobile technologies to monitor chronic diseases outside traditional clinical settings, but also underscores the importance of interpretability in deep learning models to ensure clinical relevance and trust among medical professionals. In parallel, this community has explored methods to ensure privacy and security in HAR systems. Melanson et al. [103] address the challenges of data sensitivity in personalized HAR by implementing secure multi-party computation techniques. Their approach enables the collaborative training of models across distributed data sources without compromising user privacy—an essential consideration for real-world deployments of HAR systems. Furthermore, Stojchevska et al. [104] assess the transition of HAR systems from controlled lab environments to real-world settings, emphasizing the importance of personalization in achieving reliable performance. Their findings highlight that HAR models tailored to individual users demonstrate enhanced effectiveness, thus reinforcing the value of adapting algorithms to specific behavioral patterns and contexts. Together, these studies reflect this community’s commitment to translating HAR technologies from theoretical constructs to practical, ethically grounded, and personalized applications in healthcare and daily life.

The fourth community has made a lot of progress in researching user-centered methods for transfer learning and the automatic adaptation of smart home automation systems. Most of this work has been done by academics from the UK. To avoid having to do a lot of manual setups and make it easier for people to use technology, this working group is based on the idea that one of the biggest problems with building smart homes is that systems need to be able to learn from new users who have different profiles, behavior patterns, and preferences. Ali, Augusto, and Windridge’s (2019) thorough assessment is important because it sets the stage for future progress by bringing together different methods and strategies that are meant to make intelligent and adaptable home environments more personal [105]. This community’s plan goes beyond just changing the surface. It uses the latest techniques from machine learning and artificial intelligence to let the system automatically adapt to each user’s specific needs. Du, Farrahi, and Niranjan (2019) and Ye (2018), for example, work on cascaded neural network architectures and shared learning of human activity labels across multiple datasets [106,107]. This is to make it possible for systems to share information between users and different situations. These contributions are necessary for improving model generalization and reducing dependence on extensively labeled data in every new scenario. Spathis et al. (2021) also expanded this framework by incorporating self-supervised learning techniques to derive physiological representations from data collected in real-world contexts through wearable devices [108]. These methods are necessary for enabling systems to better understand user conditions and actions without requiring intrusive interventions. Shah et al. (2019, 2022) utilized micro-Doppler radar technologies for human activity recognition and fall detection across diverse environments, illustrating the concurrent exploration of data portability and system interoperability within the community [78,109]. This enhances the applicability of these methodologies in both everyday scenarios and critical contexts such as healthcare [78,110]. Recent studies, such as Ghayvat and Gope (2023), use these ideas to smartly keep an eye on older people and find dementia early on [110]. These studies demonstrate the integration of adaptive models and user-centered methodologies into preventive wellness and care frameworks for at-risk populations [110]. Overall, this body of work sets this community as a model for building smart systems that not only adapt to new users but also improve their quality of life by providing personalized, non-intrusive, and predictive solutions. In general, the research done by this community shows a strong commitment to making smart environments that are really user-centered, can respond to different situations, and keep learning. This field of work not only makes it easier to set up devices, but it also opens the door to a more proactive and intuitive home experience. It could be used for anything from home automation to preventative health and active aging.

Under the guidance of primarily British researchers, the fourth community has made substantial progress in the study of user-centered approaches for transfer learning and the automatic adaptation of smart home automation systems. This working group is based on the notion that one of the main challenges in the development of intelligent homes is the ability of systems to learn from new users with heterogeneous profiles, behavior patterns, and preferences. This will help to avoid extensive manual configurations and reduce friction in the adoption of technology. In this regard, the comprehensive analysis by Ali, Augusto, and Windridge (2019) is important because it establishes the foundation for further developments by integrating different methods and techniques intended to personalize the experience in smart and flexible home settings [105]. The approach used by this community goes beyond superficial personalization. It uses state-of-the-art techniques from artificial intelligence and machine learning to allow the system to automatically adapt to the particular needs of each user. For instance, the work of Du, Farrahi, and Niranjan (2019) and Ye (2018) focuses on cascaded neural network architectures and shared learning of human activity labels across many datasets to allow computers to transmit knowledge across users and different scenarios [106,107]. These contributions are crucial for improving model generalization and reducing dependence on highly labeled data for every new scenario. Furthermore, by incorporating self-supervised learning techniques to derive physiological representations from data collected in real-world scenarios utilizing wearable devices, Spathis et al. (2021) expanded this approach [108]. These techniques are crucial for improving systems’ understanding of user conditions and behavior without requiring intrusive interventions. Shah et al. (2019, 2022) have shown how the community has simultaneously explored data portability and system interoperability, expanding the applicability of these approaches in both routine situations and critical contexts like healthcare [78,109]. Their studies use micro-Doppler radar technologies to address human activity recognition and fall detection in a variety of environments. More recent research, like the one by Ghayvat and Gope (2023), apply these concepts to the intelligent monitoring of aging and the early detection of dementia in older adults [110]. These research demonstrate how to include user-centered techniques and adaptive models into preventive wellness and care scenarios for people at risk [110,111,112]. All things considered, this body of work positions this community as a benchmark for developing intelligent systems that offer non-intrusive, customized, and predictive solutions that not only adapt to new users dynamically but also enhance their quality of life. Overall, the research conducted by this group demonstrates a strong commitment to developing intelligent environments that are really user-centered, able to respond contextually, and capable of ongoing learning. In addition to reducing the time and effort required to configure the devices, this field of work opens the door to a more proactive and user-friendly domestic experience, with possible applications ranging from home automation to preventative health and active aging.

Finally, Finland and Algeria form the sixth community, where a multi-modal vision-based framework for action recognition is described. This study represents an advancement at the intersection of computer vision and multi-modal signal processing, focusing on how to improve the accuracy and robustness of human action recognition [113]. The multi-modal framework proposed by the authors uses visual data combined with other types of signals to identify and classify specific actions. This approach is known to be richer and more descriptive compared to systems that rely solely on one type of signal, as it combines information from different sources to gain a more complete understanding of the activity being observed. The researchers address the technical challenges associated with synchronizing and fusing different types of data and propose algorithms for the efficient and effective processing of these combined signals. Furthermore, the study exploresthe application of deep learning representations to extract relevant features from multi-modal data and how these representations can be utilized to train action classification models.

### 5.2. Leading Journals in Human Activity Recognition Publications

This section provides essential guidance for researchers interested in maximizing the visibility and impact of their publications in the field of HAR. By detailing the leading journals and conferences that stand out in HAR publications, researchers can identify the most suitable platforms to disseminate their findings and gain greater recognition in the scientific community. Additionally, the analysis helps identify key trends and emerging advances in the field by highlighting which journals publish high-impact research. This information not only assists researchers in selecting the most influential publications for their studies but also highlights the journals that lead the development of new methodologies and applications in HAR, thereby facilitating progress in knowledge and innovation in this technological area.

Table 10 provides a clearer representation of the academic relevance of publications on HAR. The table distinguishes between articles indexed simultaneously in both databases (WoS and Scopus) and those indexed exclusively in one of them, thereby avoiding redundancy and improving the precision of the bibliometric analysis. The impact factor and quartile values reported correspond primarily to the SJR (Scimago Journal Rank) indicators for journals indexed in Scopus, while Journal Citation Reports (JCR) metrics are included when available for journals indexed in Web of Science. Additionally, the ICORE ranking is incorporated as a complementary evaluation criterion for conference proceedings, particularly for venues that do not have traditional impact factor metrics. This approach strengthens the completeness and transparency of the scientific assessment within the field.

- Sensors has 18 publications indexed in WoS and 14 in Scopus. With an impact factor of 0.76 and an H-index of 219, it is positioned in Q1, indicating it is among the top 25% of journals in its category. Notable contributions include those by author Fu [96], who provides a significant contribution to the field of wearable sensors and HAR, presenting a model that combines advances in sensor technology and machine learning methods. This work has the potential to significantly improve the efficiency and effectiveness of health and wellness monitoring systems, contributing to a better understanding and support of people’s daily lives, as well as the prevention and management of health conditions.
- Lecture Notes in Computer Science (Including Subseries Lecture Notes in Artificial Intelligence and Lecture Notes in Bioinformatics) has no publications indexed in WoS but 14 in Scopus. Its impact factor is 0.32, and it has a high H-index of 446, placing it in Q3. Relevant contributions include those by author Negi [114], who explores the use of predictive analytics for human activity recognition using a residual network (ResNet) and fine-tuning techniques. This study focuses on improving HAR through deep learning algorithms, a fundamental task in areas such as digital health, security, and sports.
- IEEE Internet of Things Journal shows a balance between WoS and Scopus with 7 publications in each database. It stands out with an impact factor of 3.75 and an H-index of 149, also positioned in Q1. Author Wang [115] demonstrates how his method can be effectively applied to HAR in different contexts, using multimodal data that includes both CSI signals and other sensors when available. The proposed approach significantly improves recognition performance compared to traditional methods, offering a more flexible and robust solution for HAR in the Internet of Things (IoT). This study contributes significantly to HAR and IoT research, providing an advanced methodology for the accurate interpretation of human activities in smart environments. The application of GANs for CSI data analysis opens up new possibilities for developing monitoring and assistance systems without the need for wearables or cameras, which is especially valuable in health, security, and home comfort applications.
- ACM International Conference Proceeding Series has no entries in WoS but has 7 in Scopus and an impact factor of 0.21. Its H-index is 137. Wang is a representative author exploring the use of transferred deep learning for cross-domain activity recognition [101]. This study addresses one of the key challenges in HAR: how to improve the generalization of deep learning models to new domains or contexts that were not seen during training. To address this issue, the authors propose a transferred deep learning approach that adapts to new domain conditions without requiring extensive domain-specific labeled datasets. This is achieved by fine-tuning pre-trained models in a data-rich source domain to a data-scarce target domain, allowing the model to retain the generalizable knowledge learned from the source domain while adapting to the peculiarities of the target domain.
- Neural Computing and Applications is not present in WoS but is in Scopus with 6 publications. It has an impact factor of 1.17 and an H-index of 111, placing it in Q1. Among the most notable works are those by author Ozcan [69], who focuses on developing advanced hand gesture recognition systems through the application of convolutional neural networks (CNNs) based on transfer learning, complemented by heuristic optimization techniques. This research is positioned at the intersection of computer vision and artificial intelligence, aimed at improving the interface between humans and computers through the accurate interpretation of hand gestures, an area of growing importance for applications ranging from healthcare assistance to device control and augmented reality. Ozcan and Basturk go further by integrating heuristic optimization techniques into the transfer learning process to efficiently adjust CNN model parameters to the specific task of gesture recognition. Heuristic optimization refers to the use of approximate methods to find optimal or near-optimal solutions to complex problems, often with fewer computational resources than exact optimization methods. In this context, it is used to refine the adapted CNN models, improving their ability to recognize hand gestures with high accuracy.
- Computers, Materials & Continua is indexed in Scopus with 5 publications but does not appear in Web of Science (WoS). The journal has an impact factor of 0.53 and an H-index of 51, placing it in the Q2 quartile. Authors like Kiran [116] address the topic of human action recognition through the fusion of deep multilayer features obtained through deep learning. This study introduces a novel approach to improving the accuracy and efficiency of human action recognition in videos, a research area of great importance for applications ranging from security surveillance to human-machine interaction and sports analysis. The approach proposed by Kiran and co-authors uses convolutional neural network (CNN) and recurrent neural network (RNN) models to extract and analyze the visual and temporal features of videos, respectively. These models are known for their ability to learn complex and highly discriminative data representations. The key innovation of the study lies in how these multilayer and model features are combined to form a unified representation that is more representative and robust for action recognition.
- Expert Systems With Applications is indexed with 4 entries in Web of Science (WoS) and 3 in Scopus. The journal has an impact factor of 1.87 and a high H-index of 249, positioning it in the Q1 quartile. Bermejo [117] introduces an innovative methodology for real-time change point detection, with a particular focus on activity segmentation within time series data collected in smart home environments. This approach is based on embedding techniques to effectively analyze and process large volumes of data generated by various devices in the smart home, with the aim of identifying significant changes in activity patterns that may indicate transitions between different activities or events. The article stands out for its proposal of a model that integrates embedding techniques to transform time series data into a feature space where change points can be detected more efficiently. This approach overcomes challenges associated with the variability and complexity of data in real-world environments, improving the accuracy of change point detection compared to traditional methods.
- IEEE Access has 5 publications in both WoS and Scopus, an impact factor of 0.93, and an H-index of 204, also in Q1. Abdulazeem [118] explores a transfer learning-based approach for human action recognition. This work addresses the challenge of identifying and classifying various human activities through visual data, a fundamental research area for developing applications in security, surveillance, sports analysis, and human-computer interaction systems. The research presents how pre-trained convolutional neural networks (CNNs) can be adapted for HAR, fine-tuning the models to specific human action datasets with a relatively small amount of training data. This approach not only reduces the need for large volumes of data specifically labeled for HAR but also decreases the time and computational resources required for model training. The results obtained by Abdulazeem and his co-authors demonstrate that the transfer learning approach significantly improves the accuracy of human action recognition compared to methods that do not use knowledge transfer. This validates transfer learning’s effectiveness as a powerful strategy for addressing HAR challenges, facilitating the implementation of more efficient and accessible recognition systems.
- Proceedings—International Symposium on Wearable Computers, ISWC has no publications in WoS but has 5 in Scopus and no impact factor or quartile, although it has an H-index of 57. Du [106] makes important contributions focused on applying transfer learning techniques for human activity recognition across different contexts, employing an innovative cascade neural network architecture. This study addresses a crucial challenge in HAR: how to improve machine learning models’ ability to generalize and adapt to new activities or users not seen during the training phase. To overcome this challenge, the authors propose a cascade neural network architecture that facilitates knowledge transfer between related but distinct HAR tasks. This approach allows the model to learn general features of human activities in an initial stage and then apply this knowledge to specific recognition tasks in subsequent stages, adjusting and refining its predictions.
- Communications in Computer and Information Science does not appear in WoS, has 4 in Scopus, an impact factor of 0.19, and an H-index of 62, ranking in Q4. Among the most relevant authors is Palak [119], who addresses the challenge of recognizing human actions from static images. This study represents a valuable contribution to the field of computer vision, focusing specifically on how advanced techniques can be used to interpret and classify various human actions captured in photographs, an area that has attracted increasing interest due to its applications in security, surveillance, multimedia content analysis, and human-machine interaction systems. The study proposes a methodology that combines deep learning techniques with a detailed analysis of human body posture and orientation, allowing the model to distinguish between a wide range of human activities. This involves using convolutional neural networks (CNNs) for feature extraction and analyzing the spatial configuration of the body, leveraging the CNNs’ ability to learn high- and low-level representations of visual data.

**Figure 4:**
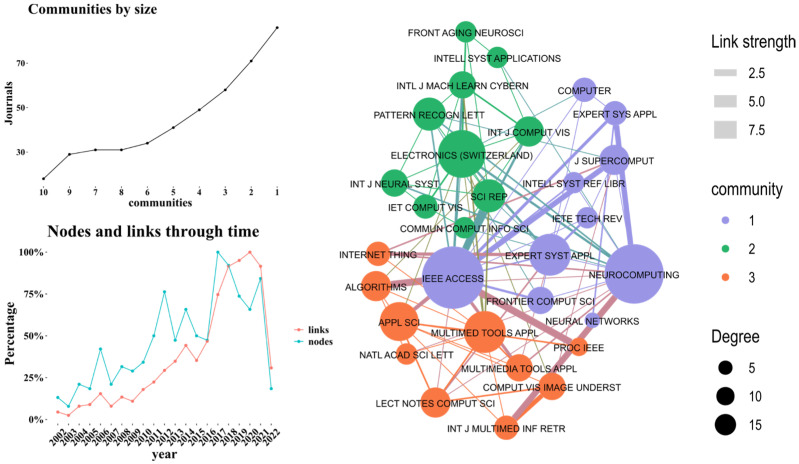
Analysis of Citation Network of High-Impact Journals.

In the citation map among high-impact journals in the field of human action recognition, a first community (shown in purple in Figure 4) can be identified, led by IEEE Access and closely linked to Neurocomputing. This community has contributed to the development of activity recognition and anomaly detection systems in smart environments. One of the reference works proposes a transfer learning-based strategy to improve model performance in data-scarce scenarios and enhance their generalization to new domains [118]. Additionally, an advanced reinforcement learning algorithm, such as Double Deep Q-Learning (DDQL) with Prioritized Experience Replay (PER), has been used to improve anomaly detection in smart homes and offices. This technique enables more refined sequential decision-making, adapting in real time to changing environmental conditions and favoring the most informative experiences to accelerate the agent’s learning [120]. Other works by this community include a comparison of various human detectors and CNN feature extractors on drone-recorded videos. These studies provide objective metrics for selecting the best combination of architectures in complex aerial-view environments [121]. Finally, human action recognition in resource-constrained scenarios has also been addressed through shared bandpass filters trained with deep learning to improve the efficiency and accuracy of models deployed on low-power devices [122].

The second-ranked publication community, led by Electronics, focuses on methodological innovation to identify human actions in smart environments. An outstanding method in this direction suggests using language models, which are often employed in natural language processing (NLP), as a tool to analyze information obtained from environmental sensors distributed throughout the home. This strategy makes it possible to model the sequence of events detected by the sensors as if they were “sentences,” where each “word” is a specific sensory event, and then apply natural language processing methods to understand these sequences and distinguish the human activities they describe [123]. In this same community, assistance systems for individuals with neurodegenerative diseases such as Alzheimer’s are being developed, employing deep learning architectures that can adapt to users’ behavior at home. This fosters greater autonomy and safety [124]. Additionally, a systematic analysis was conducted on the use of computer vision-based transfer learning for human activity recognition, evaluating how these methods enable reduced reliance on large amounts of labeled data and optimize adaptability to new users or contexts [125]. Finally, a study is highlighted that focuses on the decision-making process of when and why to transfer knowledge between different individuals within activity recognition systems, using a transferred meta-learning method. This work contributes not only to increasing accuracy but also to improving the controllability and interpretability of the models, enabling a better understanding of the system’s decision-making processes [126]. Overall, this community makes a significant contribution to the convergence of assistive technologies, natural language processing, and artificial intelligence, strengthening perspectives that are highly interpretable, customizable, and focused on the end user.

The third community is represented by the works published in Multimedia Tools and Applications, and its objective is to identify human actions using still images and other multimedia formats. One of the most important contributions addresses this task through fine-tuning-based transfer learning methods, which are implemented on pre-trained convolutional neural networks. This method enables the model to effectively adapt to the specific characteristics of human actions captured in images, thereby increasing accuracy without requiring training from scratch. This is an efficient solution in terms of computing resources and the time required for processing [127]. On the other hand, a different study from the same community suggests converting videos into images. This technique, applied to skeletal data, enables a detailed analysis to identify human activities. This strategy aims to capture the body structure and fundamental movements of individuals, enabling a robust representation of activity even in challenging video conditions [128]. Additionally, a method employing sensors and transfer learning has been investigated to identify human actions in various locations. This vertical data distribution of the model enables recognition across different sensor positions to generalize more effectively, which is particularly beneficial in real-world situations where the location changes [129].

### 5.3. Leading Authors in Publications on Human Activity Recognition Using Transfer and Reinforcement Learning

Table 11 shows the most prominent authors leading this line of research and summarizes, in a comparative format, the scope and impact of their scientific output. Important indicators are included, such as the total number of published articles—taking into account those indexed in highly rigorous international databases such as Web of Science (WoS) and Scopus—as well as established academic impact metrics. Productivity and the number of citations received by each author are shown in the h-index, while the “Total articles” column includes the combined count of indexed publications, with an asterisk indicating cases where figures from both databases are combined. Overall, these data enable the identification of the most experienced experts and the significance of their contributions within the broader framework of this research area.

1. Chen, Y. [130], from National Taipei University, Taipei, Taiwan, with 9 articles and an h-index of 25, shows a balanced combination of productivity and considerable oimpact in his field, suggesting that his works are well recognized and cited in the scientific community, among which stands out the approach to an innovative challenge in the field of activity recognition using mobile and wearable devices. In this study, the authors focus on improving the ability of systems to recognize human activities regardless of the position of the device on the user’s body, which represents a significant problem in the practical application of activity recognition technologies in daily life.
2. Khan, M. [131], from Kennesaw State University, Kennesaw, USA, stands out notably with 8 articles but an exceptional h-index of 112. This high h-index indicates that Khan M is a highly influential researcher whose work has had a profound and widely recognized impact on his discipline, in which he has published works focused on the emerging field of the Internet of Things (IoT) and its application in human activity recognition. This author addresses a particularly complex challenge in this field: the ability to recognize activities that have not been previously seen during the training phase of the model, using unlabeled data and transfer learning.
3. Roy, N. [68], from the University Of Maryland, Baltimore County (UMBC), Baltimore, USA, with 8 publications and an h-index of 20, proves to be a researcher whose work has a respectable recognition and a significant influence, reflecting the quality and importance of his research. Among his most notable works is an innovative approach to human activity recognition through the use of transfer learning. This work addresses the problem of how to improve the efficiency and effectiveness of human activity recognition (HAR) systems in situations where datasets are limited, diverse, or where data collection conditions vary significantly. “TransAct” proposes a transfer learning framework specifically designed for HAR, with the aim of overcoming the limitations of traditional approaches that require extensive amounts of labeled and context-specific data to train accurate models. Transfer learning, in this context, is used to transfer knowledge gained from an activity recognition task (with a large, well-labeled dataset) to another task where the data may be sparse, unlabeled, or where activities may vary slightly in execution due to differences in the environment, user, or data collection device.
4. Qin, J. [95], affiliated with Microsoft Research Redmond, USA, with 8 papers and an h-index of 21, shows an important contribution to his field. His affiliation suggests a privileged position to combine academic research with practical applications in industry. The author introduces an advanced methodology for activity recognition using spatial-temporal adaptive transfer learning. This study addresses one of the most significant challenges in the field of human activity recognition (HAR), especially in environments characterized by data heterogeneity among different datasets. The main objective of the work is to improve the ability of HAR systems to generalize across multiple datasets without the need for extensive recalibration or relabeling. This is particularly relevant in Internet of Things (IoT) applications and wearable devices, where the diversity of devices, users, and contexts can lead to high variability in the data collected.
5. Zebhi, S. [132], from Yazd University, Yazd, Iran, with 6 publications and an h-index of 12, shows that although he has fewer publications than others on this list, his work is of quality and has gained a respectable number of citations, showing its impact on the academic community, as he addresses the task of human activity recognition (HAR) through an innovative technique that uses human motion images (MHIs) generated from sequences of frames. This work is enclosed in the field of computer vision and artificial intelligence, offering a novel methodology to improve the accuracy and efficiency of HAR, a crucial area of research for applications in security, health, and interactive systems. The method proposed in the article is based on the generation and analysis of MHIs from sequences of video frames to identify and classify different human activities. The authors develop and apply advanced algorithms to process these MHIs, extracting key features that allow the accurate identification of the activity performed. Through this approach, the study seeks to overcome some of the limitations of traditional HAR methods, which often require complex preprocessing or cannot effectively handle variability in activity execution.
6. Oh, S. [86], from Inha University Incheon, South Korea, has 6 papers and an h-index of 3. This profile suggests that Kim Y is possibly a newer researcher or that his work is just beginning to gain recognition in his area of study. The author focuses on an advanced methodology to improve the accuracy and efficiency of HAR by using semi-supervised learning and active transfer learning. This study addresses critical challenges in HAR, such as label sparsity and data diversity in practical applications, by proposing an innovative solution that combines the advantages of semi-supervised learning and active learning to optimize the use of unlabeled and labeled data effectively. The research introduces an active semi-supervised learning framework in which a model initially trained with a small labeled dataset is iteratively improved by actively selecting and labeling the most informative samples from an unlabeled dataset. This strategy allows the system to efficiently identify the samples that, once labeled, will contribute most significantly to model improvement. Combined with transfer learning techniques, this approach allows pre-trained models to be adapted to new HAR contexts or tasks with minimal additions of labeled data, thus overcoming the limitations of purely supervised or unsupervised approaches.
7. Myagmar, B. [133], from Harbin Institute Of Technology, Harbin, China, with 6 publications, where he stands out for addressing a central problem in the field of ADL recognition through big data analysis: the heterogeneity of data from different domains. This study proposes an innovative solution to effectively learn about heterogeneous ADLs by identifying and leveraging a domain-invariant feature subspace, thus improving the generalization of machine learning models in ADL recognition tasks.
8. Li, X. [134], from the University Of Technology Sydney, Sydney, Australia, with 6 papers and an h-index of 7, shows a moderate impact in his field, with a balance between the amount of work produced and the recognition obtained through citations. The author introduces a state-of-the-art methodology for human activity recognition using micro-Doppler signatures obtained through radar technology. This research is situated at the intersection of remote sensing and machine learning, proposing a semi-supervised approach to identify human activities, which is critical for applications ranging from security and surveillance to healthcare and disaster management.
9. Zebhi, S. [135], also from Yazd University, Yazd, Iran, with 6 publications and an h-index of 3, shares a similar profile to Kim Y, suggesting that they are either in the early stages of their academic careers or that their research areas are emerging in terms of recognition and citations. The authors address the challenge of HAR by using pre-trained neural networks and implementing informative templates. This approach is proposed as an innovative solution to improve the accuracy and efficiency of HAR, especially in scenarios where collecting and labeling large volumes of activity-specific data may prove prohibitively expensive or impractical. To overcome these limitations, Zebhi and his co-authors propose a method that leverages pre-trained neural networks, which have already learned useful features from large datasets in general computer vision tasks. The key innovation of their approach lies in the integration of these networks with informative templates specifically designed to highlight the most relevant features for activity recognition. These templates act as a filter that guides the pre-trained network to focus on particular aspects of the input data that are most informative for distinguishing between different activities.
10. Finally, Almodarresi, S. [136] is the last author who focuses on identifying the use of transfer learning applied to human activity recognition (HAR) through spatiotemporal representations. This work addresses the challenge of how to improve the accuracy and generalization of HAR systems in diverse environments and conditions, using an innovative approach that combines transfer learning with the detailed analysis of movement patterns in time and space. The authors propose a framework that uses transfer learning to adapt HAR models pre-trained in a source domain to a new target domain, with an emphasis on capturing and analyzing spatiotemporal representations of activities. These representations allow capturing the dynamics of human movement more completely, considering both the spatial distribution of movements and their evolution over time.

**Figure 5:**
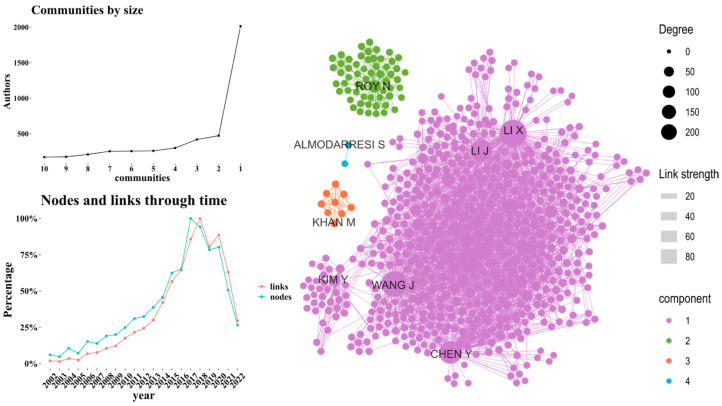
Analysis of Citation Network of Authors.

Similarly, these global research leaders have created a scientific synergy that is reflected in four relevant communities (see Figure 5). The purple community, composed of Asian authors such as Liu J., Liu X., Wang J., Kim Y., and Chen Y., is characterized by driving novel research in the field of HAR using deep learning techniques. For human detection via wireless means, a specific federated learning framework has been proposed that enables model exchange across diverse domains without requiring the sharing of sensitive information. This enables collaborative learning in sparse detection scenarios [137]. This proposal is based on adaptive models, such as AdaRNN, which can predict and learn complex time series through attention and transfer mechanisms. This enables more accurate recognition in changing circumstances [83]. Additionally, stratified transfer learning methods have been developed to address the problem of activity recognition across different body postures, representing a significant advance in generalizing models trained in diverse contexts [138]. Other studies, along the same lines, investigate cross-dataset recognition through adaptive learning in temporal and spatial terms, which optimizes the model’s ability to adapt its predictions to new data collection circumstances [96]. Finally, a method based on substructural optimal transport is presented, which promotes cross-domain adaptation by aligning the latent structures present in the data. This allows the system to better distinguish human activities in contexts with different distributional properties [100].

With strong leadership from the United States, the second and third communities have notable representatives such as Faridee, Roy, and Khan, who have suggested innovative perspectives in the field of HAR. One of the main contributions is addressing the recognition of activities that had not previously been observed, a crucial problem for the practical application of HAR systems in changing and dynamic contexts. Along these lines, Alam and Roy propose a hierarchical active transfer learning method; this approach enables models to recognize new activities by using unlabeled data and a decision hierarchy that improves the adaptation process [139]. This line is complemented by the UnTran proposal, which uses transfer learning methods to identify unknown activities without requiring labeled data from the domain in question, leveraging the generalization of what has been learned from previous activities [140]. Additionally, Khan and Roy introduce TransAct, a framework that enables the scalability of HAR through transfer methods that adapt pre-trained models to new domains [68]. Khan et al. address the challenge of cross-domain variability in the field of wearable devices by employing multi-source adaptation with adversarial learning. To this end, they train models that can effectively generalize to previously unseen domains without requiring large amounts of labeled domain-specific data [5]. Faridee et al. present AugToAct, a model that combines deep learning and data augmentation methods to improve system accuracy, enabling the recognition of complex activities from a small number of labels [140]. Ramamurthy and Roy, in conclusion, present a detailed analysis of the latest trends in machine learning applied to HAR, providing a broad perspective on the progress and challenges in this field [2].

The fourth community identified in this line of research is led by Almodarresi from Iran. This community stands out for proposing a revolutionary method for identifying human activities from videos, which consists of classifying image templates using a dual-stream system and pretrained neural networks. This perspective is proposed as a response to the inherent challenges in video data processing, particularly the need to capture the complexity of spatial and temporal dynamics. Within this framework, a fusion strategy is suggested that combines parallel streams to examine spatial (appearance) and temporal (motion) features by classifying templates extracted from video sequences [138]. The application of enhanced spatiotemporal representations through transfer learning methods has made it possible to optimize accuracy without having to train from scratch [141]. A subsequent study shows that combining the two-stream architecture with pretrained deep networks significantly improves performance in video classification tasks, particularly in situations where data is scarce [142]. Likewise, architectural frameworks that employ global descriptors and pretrained deep learning have been studied to recognize complex actions in videos, even when resolution, lighting, or background vary [143]. Finally, a method focused on knowledge transfer for HAR is presented. It uses models that have been trained on large volumes of data and effectively adapts them to specific tasks, reducing training time and optimizing generalization [143].

## 6. Discussion: Frontier of Knowledge in the Line of Research

**Figure 6:**
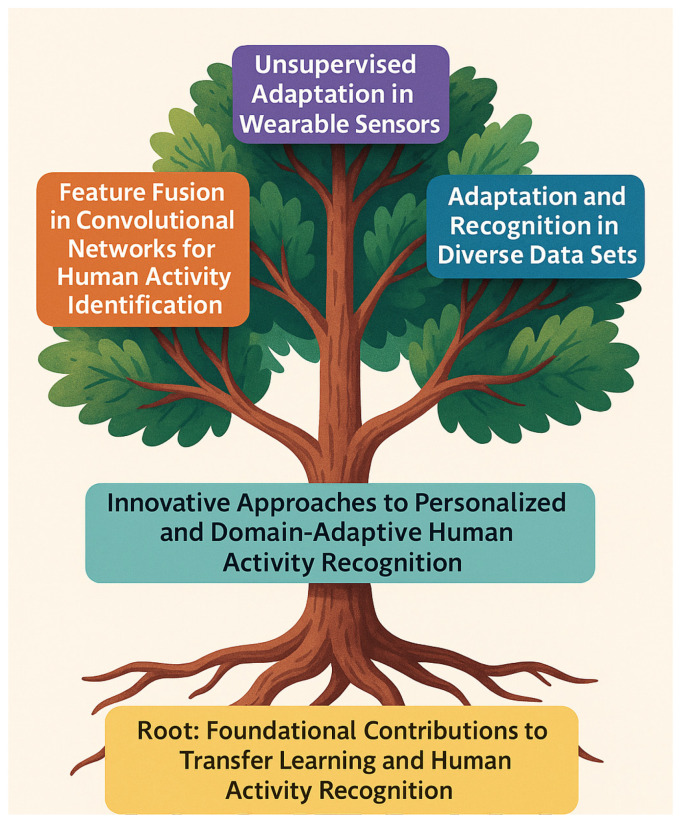
Tree of Science Representation.

By utilizing the metaphor of the “Tree of Science,” we can trace the evolution of research in Transfer and Reinforcement Learning as Support Paradigms for Human Activity Recognition, see Figure 6. In this context, attention is directed to the roots of the tree, representing the early stages when these learning paradigms faced significant challenges due to limited technological adoption and integration within the field. This phase highlights the initial hurdles encountered in applying advanced learning techniques to human activity recognition, setting the foundation for later developments.

### 6.1. Root: Foundational Contributions to Transfer Learning and Human Activity Recognition

Pan [144] shows a comprehensive guide to transfer learning, providing an in-depth analysis of its methodologies, challenges, and applications. The authors categorize transfer learning approaches into various types, such as inductive, transductive, and unsupervised transfer learning. The paper also examines how these approaches can overcome the limitations posed by insufficient labeled data in various domains. By analyzing different transfer learning scenarios, this paper establishes a foundational framework that has informed and guided subsequent research. The survey not only underscores the potential of transfer learning in improving model performance but also identifies key challenges such as negative transfer, domain divergence, and the need for effective transfer strategies. This paper has been instrumental in advancing research in fields like computer vision, natural language processing, and human activity recognition by promoting a deeper understanding of how knowledge can be transferred between tasks with different distributions.

Cooks [145] explores how transfer learning techniques can address the challenges associated with domain adaptation in HAR, such as variations in sensor placement, different environments, and individual differences among users. The survey covers a broad range of transfer learning approaches, including instance-based, feature-based, and model-based methods, and examines how these techniques can be applied to improve the robustness and accuracy of HAR systems. By providing a thorough review of transfer learning applications in HAR, this paper emphasizes the importance of adaptability in real-world scenarios, where collecting labeled data for every possible situation is impractical. The authors also discuss the future directions of transfer learning in HAR, such as the need for more sophisticated models that can handle complex activities and dynamically adapt to new users and contexts.

Bulling [146] provides detailed guidance on sensor placement, signal processing, feature extraction, and classification techniques, making it an essential resource for both researchers and practitioners. The paper outlines various challenges in HAR, such as sensor noise, variability in human motion, and the need for real-time processing. It also presents best practices for designing HAR systems, including the selection of appropriate sensors, the use of machine learning algorithms, and the integration of contextual information to improve activity recognition accuracy. By covering both the theoretical and practical aspects of HAR, this tutorial helps bridge the gap between research and application, enabling the development of robust and efficient HAR systems that can be deployed in real-world settings. The authors also highlight emerging trends in HAR, such as the use of deep learning and the incorporation of additional sensor modalities, paving the way for future innovations in the field.

### 6.2. Trunk: Innovative Approaches to Personalized and Domain-Adaptive Human Activity Recognition

Mazankiewicz [147] introduces a cutting-edge method to achieve incremental real-time personalization in HAR systems through domain-adaptive batch normalization. Traditional HAR models often struggle to generalize across different users due to individual variability in activities. The authors address this challenge by implementing a domain-adaptive batch normalization technique that adjusts the model parameters incrementally as new user data becomes available. This method allows the HAR system to adapt to the unique characteristics of each user in real-time without the need for a full retraining process. The key innovation of this method lies in its capacityto personalize the HAR model dynamically while maintaining high accuracy across different domains. This approach is particularly beneficial for applications where continuous monitoring and adaptation are required, such as in personalized healthcare or fitness tracking. The study also emphasizes the importance of reducing computational overhead during personalization. By focusing on batch normalization as the primary mechanism for adaptation, the authors ensure that the system remains efficient and scalable. The real-time aspect of this methodology is essential for practical deployment in wearable devices and mobile applications, where resources are often limited, and the need for instantaneous adjustments is critical.

Chen [148] explores the application of transfer learning in HAR systems specifically designed for smartphones. The key contribution of this paper is the development of a feature matching and instance reweighting strategy that enhances the transferability of HAR models across different users and devices. Traditional HAR systems often face difficulties when deployed in new environments due to differences in sensor data caused by variations in user behavior or hardware configurations. The authors propose a method that aligns feature distributions between the source and target domains by employing feature matching techniques. Additionally, they introduce instance reweighting, a process that adjusts the importance of each instance in the training dataset based on its relevance to the target domain. This dual approach not only improves the accuracy of HAR models when transferred to new devices or users but also reduces the need for extensive retraining on new data. The significance of this work lies in its potential to make HAR systems more adaptable and robust in real-world applications. With the increasing reliance on smartphones for activity monitoring, this study provides valuable insights into overcoming the challenges associated with domain adaptation in HAR.

Zhang [149] explores the use of radar technology for HAR in elderly care, addressing the limitations of traditional methods like cameras and wearables. Radar-based HAR offers several advantages, including non-invasiveness and the ability to operate in environments where privacy is a concern, making it particularly suitable for monitoring elderly individuals in their homes. The paper presents a novel approach that combines radar data with hybrid maps to improve the accuracy of activity recognition. By leveraging an open dataset, the authors demonstrate the effectiveness of their method in classifying various activities relevant to elderly care, such as detecting falls or monitoring daily routines. The study also highlights the importance of using hybrid maps, which integrate radar signals with contextual information, to enhance the model’s ability to differentiate between similar activities. The implications of this research are significant for the field of elderly care, as it provides a scalable and privacy-preserving solution for continuous monitoring. The use of radar technology, coupled with advanced machine learning techniques, offers a promising direction for future developments in HAR systems aimed at improving the quality of life for elderly individuals while maintaining their independence.

### 6.3. Branch 1: Feature Fusion in Convolutional Networks for Human Activity Identification

The frontier feature fusion in convolutional networks for human activity identification is rapidly expanding, pushing the boundaries of what is possible in activity recognition using wearable sensors. This line of work is deeply influenced by advances in deep feature optimization, inertial data analysis, and personalization through transfer learning. On the one hand, authors such as Sahoo et al. [73] have notably contributed to the development of wrapper methods for deep feature optimization specifically in wearable sensor networks for healthcare systems. Their research in “Scientific Reports” highlights how feature optimization techniques can improve the selection of relevant features, which is crucial for the effectiveness and efficiency of activity recognition in healthcare applications. This approach is a significant advancement, as it addresses the dimensionality and relevance of data in contexts where accuracy is vital to clinical outcomes.

On the other hand, Gomaa [150] and Khamis provide a fresh perspective on human activity recognition using inertial motion data in “Neural Computing and Applications.” They explore how data fusion from inertial sensors, such as accelerometers and gyroscopes, can be effectively analyzed to capture the subtleties of human motion. Their focus on signal processing and temporal and frequency feature extraction is setting new standards in activity recognition accuracy and underscores the importance of understanding the nature of motion as a temporal sequence.

Finally, Bursa, Incel, and Alptekin [151] advance personalization in activity recognition with their paper in “Computers and Electrical Engineering.” They use transfer learning coupled with compressed deep learning models to address inter-person variability in activity performance. Their approach to personalization, which adjusts general models to individual peculiarities, is especially promising for applications requiring high customization, such as in personalized healthcare and elderly care systems. The convergence of these three bodies of work represents the cutting edge of HAR research: the fusion of deep optimized features, refined processing of inertial data, and personalization through transfer learning. These collective strategies are defining the next generation of activity recognition systems that will be more accurate, adaptive, and capable of operating in real-time, which has significant implications in health monitoring, rehabilitation, and user-machine interface. This is a constantly evolving field, where each new research brings us closer to a more holistic integration of technology into daily life and personalized healthcare.

### 6.4. Branch 2: Unsupervised Adaptation in Wearable Sensors

The frontier of knowledge in the domain of unsupervised adaptation in wearable sensors is marked by a series of studies seeking to improve activity recognition across individuals and sensor positions without requiring intensive manual labeling. Recent developments in this field open promising paths toward deeper personalization and adaptability in real-life applications. Shen, Teso, Giunchiglia, and Xu, in their publication in “Electronics,” have addressed the critical decision of when and why to transfer knowledge across individuals in the context of activity recognition.

Their work on meta-transfer learning not only facilitates adaptation across subjects but also improves the interpretability and controllability of activity recognition models. By focusing on the “how” and “why” of the transfer learning process, their approach enables systems to better adapt to human variability while maintaining transparency and ease of tuning. Shen [126], Xu [152], Jia [153], and Meng have integrated deep adaptation learning with online extreme sequential learning machines in Expert Systems with Applications, proposing an innovative methodology for activity recognition that transcends both individual differences and variability in the position of wearable sensors. Their approach addresses two of the main challenges in HAR: inter-person variability and sensor position dependence. Combining these two techniques improves the system’s ability to dynamically adapt, which is essential for real-time applications. Jia, Guo, Yang, and Yang, meanwhile, offer in Complex & Intelligent Systems a holistic approach using multi-source transfer learning with wearable sensors for personalization in daily activity recognition. By considering multiple data sources and bridging domain adaptation with personalization, their work enables activity recognition deeply tailored to individual needs and behavior patterns. This approach promises increased accuracy and utility in personalized activity recognition in complex and dynamic environments.

### 6.5. Branch 3: Adaptation and Recognition in Diverse Datasets

The frontier of knowledge in the fieldis advancing rapidly, driven by the development of innovative techniques and the application of deep learning approaches for the identification of complex patterns in large volumes of data. Recent research has contributed significant advances, optimizing and refining the way machine learning models can accurately recognize and classify human activities and security threats in smart grids. Nasir et al. [154] in their work presented at “Expert Systems with Applications” propose ENGA, a methodology based on genetic algorithms and elastic net for human action recognition. This pioneering approach combines elastic net regularization with genetic algorithm heuristic search, offering a powerful mechanism for selecting and optimizing features in human activity recognition datasets. ENGA focuses on improving accuracy by balancing the detection of relevant features and dimensionality reduction, which is essential for dealing with high-dimensional data and avoiding overfitting. Li, Ma, and Sun [155], explore in “Algorithms” the adaptability of deep neural networks in intrusion detection in smart grids. Their adaptive deep neural network model is designed to improve machine learning-based classifiers, demonstrating deep learning’s ability to adapt to new threats and variations in data. This work is not only relevant to critical infrastructure security but also illustrates how adaptive approaches can significantly improve the effectiveness of intrusion detection systems.

Finally, Ray, Kolekar, Balasubramanian, and Hafiane [156], in their article published in the “International Journal of Information Management Data Insights”, conduct a comprehensive decade-long analysis of how transfer learning has improved vision-based human activity recognition. Their study synthesizes advances in the field, highlighting how transfer learning has enabled the utilization of knowledge gained in one data domain to improve performance in others. This review provides critical insights into the development of human activity recognition systems that are robust to variations in datasets and capture environments. Collectively, these studies represent the state of the art in adaptation and pattern recognition across diverse conditions and datasets. Through the fusion of statistical methods, evolutionary algorithms, and deep learning, current research is pushing the boundaries toward systems that are not only highly accurate but also capable of adapting and generalizing across a broad spectrum of applications, from health monitoring to cybersecurity. These advances are crucial to the development of intelligent and autonomous technologies that seamlessly integrate into our infrastructure and everyday lives.

### 6.6. Challenges and Gaps in Transfer Learning for Human Activity Recognition

It is feasible, through a detailed review of the literature on transfer learning in human activity recognition systems, to identify not only established trends but also various methodological gaps that limit the applicability and maturity of these approaches in real-world settings. Although a large body of research demonstrates significant technical advances—in regularization, cross-domain adaptation, or architecture—there remains a lack of systematicity in the development of methodological frameworks that coherently incorporate real-world challenges, particularly in healthcare settings where conditions are inherently dynamic and diverse. The lack of proposals that address adaptive and continuous transfer learning in a robust manner is one of the most notable elements. This is fundamental for systems that need to operate in constantly changing environments, such as outpatient monitoring of patients with chronic diseases or clinical monitoring at home. Although methods such as few-shot learning or domain adaptation have been highlighted, they are often still assumed to operate under controlled conditions or on closed datasets, without taking into account factors common in clinical applications, such as human–system interaction, temporal variability, or progressive personalization.

Furthermore, there is a significant lack of explanation regarding the models used. Although the literature favors models of increasing complexity, such as hybrid architectures or deep neural networks, it does not provide methods to understand or justify their predictions. This represents a barrier to implementation in the healthcare sector, where systems must be auditable, comprehensible to experts who are not specialized in artificial intelligence, and comply with high-quality ethical and regulatory standards. Therefore, it is not only desirable but also essential to move toward solutions that incorporate principles of interpretability and explainability for their practical viability. Regarding validation in real-world healthcare settings, the results obtained indicate that there is still a significant gap between experimental studies and their practical application in clinical situations. Although certain studies have achieved partial transfers, such as monitoring older adults or detecting falls via wearable sensors, these are typically exploratory and lack longitudinal evaluations to determine their sustainability, scalability, and effectiveness in real-world settings. Above all, the clinical data ecosystem faces unique challenges: the variety of sensors, the patient’s physiological changes, the dependence on the environmental context, and privacy limitations. They all require specific and flexible methodological approaches.

Therefore, it is urgent to have a research agenda that not only seeks to improve the accuracy of models but also ensures their operational robustness, their ability to adapt continuously, and their clinical validity. This requires engineers, physicians, data scientists, and interaction designers to collaborate with one another, as well as the formation of consortia that enable access to real-world data while adhering to precise technical and ethical standards. In summary, while the advances analyzed in this study are significant and reflect important progress in algorithms and methods, the field is still in a transitional phase between academic research and practical implementation. Identifying methodological gaps and fostering their closure through applied research represents a strategic opportunity to establish transfer learning as a truly revolutionary technology in the field of digital health.

## 7. Conclusions

The understanding and structuring of knowledge on human activity recognition through transfer learning methods benefit comprehensively from the systematic analysis presented here. The detailed examination of the most recent studies facilitated the synthesis of the state of the art in this field, the identification of shared methodological patterns, the proposal of a taxonomy of approaches, and, above all, the discovery of opportunities that still lie dormant for conducting new research. The most relevant contributions are detailed below:

1. Consolidation of a theoretical framework through the “Tree of Science”

One of the greatest legacies this manuscript leaves is the “Tree of Science.” This analytical and visual tool combines, by employing transfer learning to recognize human actions, emerging trends (branches), essential contributions (roots), and core methods (trunk). The hierarchical model enables, in addition to understanding accumulated knowledge, the identification of relationships among research objectives, techniques, and fields of application. By visually incorporating the evolution of the field, the tree contributes to building a coherent scientific memory and simultaneously suggests possible directions for future research.

2. Identification of strategic thematic clusters

The review allowed the literature to be classified into three main thematic clusters (or branches), each with its own challenges and characteristics: (i) feature fusion in convolutional neural networks to improve recognition, (ii) unsupervised adaptation on wearable sensors, and (iii) robust recognition across diverse datasets. This thematic organization not only systematizes existing knowledge but also guides developers and researchers in selecting tactics based on the opportunities and constraints of the application context (for example, when personalization, real-time adaptation, or a scarcity of available labels is required).

3. Fundamental contribution to understanding current challenges

The analysis is not limited to a narrative description of the techniques; it also offers a critical examination of the field’s methodological conflicts and constraints. The need to reduce reliance on large volumes of labeled data, the importance of implementing systems that continuously adapt to changing contexts, and the urgency of improving model generalization in situations with high inter-individual variability are key points. Likewise, it is suggested that we move toward explainable models, especially in sensitive applications such as monitoring older adults or individuals with clinical conditions, as well as in health and safety.

4. Production of knowledge that can be used in practice and in science policy

Finally, the manuscript offers value beyond the theoretical realm. When evidence is compiled on the circumstances under which transfer learning improves performance, valuable insights are generated that can be used to make decisions in academic or industrial settings that rely on the automated analysis of behavioral patterns. Likewise, a research agenda is proposed to help guide collaborative, interinstitutional projects, especially in fields where the development of adaptive technologies is essential for addressing demographic and social challenges.

## Figures and Tables

**Table 1:** Type of Activities in HAR.

Conventions	Description	Technical Explanation
B	Basic or Simple Activities	These are the most common activities and involve simple, everyday movements. Examples include walking, running, sitting, standing, lying down, climbing stairs, and sleeping. These activities are often repetitive and predictable, making them easier to recognize with inertial sensors such as accelerometers and gyroscopes [8].
C	Complex or Composite Activities	These activities involve more sophisticated sequences of movements and actions. For example, preparing a meal, cleaning a room, or performing specific physical exercises require interaction with multiple objects and possibly several stages of action. Recognizing these activities is more complex due to the variability in execution and the diversity of the actions involved [9].
SC	Social or Contextual Activities	These activities greatly depend on the context and environment. For example, working in an office, attending a meeting or class, or participating in group activities. Recognizing these activities not only requires motion sensor data but also additional contextual information, such as the presence of other people, the time of execution, and the specific environment in which they occur [10].
CA	Critical or Anomalous Activities	These are infrequent but highly important activities, such as falls, fainting, or risky behaviors. Accurately detecting these activities is crucial for health and safety applications and typically requires the combination of multiple sensor types and advanced algorithms for anomaly detection [11].

**Table 2:** Sensor Modalities for Indoor Human Activity Recognition.

Conventions	Description	Technical Explanation
I	Inertial Sensors	Inertial sensors, integrated into portable devices such as smartphones, smartwatches, and fitness trackers, are essential for capturing basic human body movements. Accelerometers measure acceleration across three axes (X, Y, Z), enabling the detection of changes in speed and movement direction. Gyroscopes, on the other hand, measure rotation and orientation. These sensors are effective for simple activities such as walking or running, although their accuracy may decline when detecting complex or low-mobility activities [12].
E	Environmental Sensors	In indoor environments, environmental sensors provide contextual data that complement the information obtained from inertial sensors. Examples include temperature, humidity, atmospheric pressure, and light and sound sensors. These sensors are valuable for detecting changes in the environment that may be associated with specific activities. For instance, a light sensor can help determine whether a person is in a well-lit room (such as an office) or in a dark environment (such as a bedroom) [13].
MP	Motion and Proximity Sensors	Motion sensors, such as passive infrared (PIR) sensors, detect the presence and movement of individuals within a given area. Proximity sensors, on the other hand, can determine the distance between an object or person and the sensor. These sensors are widely used in home automation systems and security settings to monitor room occupancy and detect intrusions [14].
V	Vision Sensors	RGB cameras capture images and videos that can be processed using computer vision techniques to recognize postures, gestures, and complex movements. Depth cameras, such as those used in Kinect systems, provide three-dimensional information about body position and objects in the environment. These sensors are particularly useful for complex and context-dependent activities, where visual information is essential for accurate analysis [15].
B	Biometrics Sensors	These sensors measure physiological signals such as heart rate, brain electrical activity (EEG), respiration, and body temperature. Biometric sensors can provide additional information about an individual’s physical and emotional state during activities. For instance, an elevated heart rate may indicate intense physical exertion, while variations in brain activity may be associated with changes in attention or stress [16].

**Table 3:** Dataset for HAR in Indoor Environments.

Dataset	Description	Sensor Used	Activities Covered	Limitations/Gaps	Sensor Type(s)	Activity Type(s)
CASAS [17]	Developed by Washington State University, this dataset contains data collected from environmental and motion sensors installed in real homes, along with detailed annotations of residents’ daily activities. CASAS is widely used in research on home automation, elderly care, and indoor security.	Motion sensors (PIR), door/window sensors, temperature sensors, light sensors, water flow sensors	Daily activities: sleeping, eating, cooking, personal hygiene, work, leisure, housekeeping	Limited representation of outdoor activities and social interactions	E	B, SC
ARAS [18]	The ARAS dataset focuses on data collection in real apartments, where residents are monitored during their daily routines. It combines data from environmental sensors and wearable devices to capture a broad range of activities in indoor environments, making it particularly valuable for developing algorithms capable of recognizing activities in complex and ambiguous scenarios.	Accelerometers (wearable), gyroscopes (wearable), ambient sensors (PIR motion, pressure mats)	27 activities including: walking, sitting, lying, eating, drinking, brushing teeth, cooking	Underrepresented: fine motor activities, cognitive tasks, social activities	E, MP	B, SC
Opportunity [19]	The Opportunity dataset is used for activity recognition in simulated environments, particularly in the fields of robotics and human–computer interaction. It contains recordings of activities performed in a controlled indoor setting, captured using a variety of wearable and fixed sensors.	12 IMUs (body-worn), 12 object sensors, 8 ambient sensors (accelerometers on doors/objects)	Locomotion, postures, manipulation activities (opening doors, drinking), ADL scenarios	Limited environmental diversity (single apartment), few interaction activities	I, E, MP	B, C
MobiAct [20]	The MobiAct dataset focuses on the collection of inertial sensor data for activity recognition in indoor environments, including accelerometer and gyroscope recordings for both basic and complex activities. It is widely used in fall detection research and in the recognition of critical events.	Accelerometer and gyroscope from smartphones (worn on body)	Falls (4 types), ADLs (walking, stairs, sitting, standing, jogging, jumping), car activities	Overrepresented: fall detection activities. Underrepresented: complex household tasks, social activities	I	B, CA
REALDISP [21]	This dataset focuses on the recognition of human activities in indoor environments using wearable devices and environmental sensors. It includes data on Activities of Daily Living (ADLs) and is commonly used in applications designed to enhance the quality of life for elderly and individuals with disabilities.	9 IMUs (chest, arms, legs), environmental sensors (ambient light, temperature, PIR motion)	33 fitness/rehabilitation activities and ADLs: walking, exercises, lying, sitting, household tasks	Strong focus on physical/rehabilitation activities. Limited cognitive tasks and multi-person scenarios	I, E	B, SC
UCAmI Cup [22]	Created to support the analysis of human activity recognition in daily living tasks, the UCAmI Cup dataset contains detailed recordings of Activities of Daily Living (ADLs), collected through the UJA dataset, which was specifically designed to evaluate activity recognition algorithms. This dataset is widely used to test models aimed at enhancing independent living and support systems for individuals with specific needs.	Binary sensors (motion, door, light, temperature), some deployments include pressure mats	Morning routine, meal preparation, medication management, hygiene, entertainment	Limited to binary sensor data (lower granularity). Underrepresented: fine-grained manipulation tasks	E, MP	B, SC
PAMPA2 [23]	This paper provides a detailed overview of the data collection protocol—using three IMUs (wrist, chest, ankle) and a heart rate sensor at 9 Hz across nine participants over 18 daily activities—and establishes PAMAP2 as a benchmark in multimodal physical activity recognition.	3 IMUs (wrist, foot, ankle), heart rate sensor (PPG)	18 ADLs: walking, sitting, lying, standing, eating, drinking, cooking, cleaning, computer work	Good physiological data integration. Underrepresented: outdoor activities, social interactions	I, B	B, CA
WISDM (Wireless Sensor Data Mining) [24]	The WISDM dataset contains accelerometer and gyroscope data collected from smartphones and smartwatches during common physical activities such as walking, running, sitting, standing, and stair climbing. The data is recorded in real-world conditions using mobile devices carried in the pocket or worn on the wrist, making it well-suited for training and evaluating machine learning models for human activity recognition in realistic scenarios.	Accelerometer and gyroscope from smartphone (pocket) and smartwatch (wrist)	18 activities: walking, jogging, stairs, sitting, standing, typing, eating, teeth brushing, folding laundry	Overrepresented: ambulatory activities. Underrepresented: fine manipulation, household complex taskssks	I	B
HAPT [25]	HAPT is an extended dataset derived from WISDM that includes not only physical activities such as walking, running, and sitting, but also detailed annotations of postural transitions like sitting down, standing up, and lying down. The data is collected using smartphone-based accelerometers and gyroscopes, making it suitable for analyzing both steady-state activities and dynamic changes in body posture.	Smartphone-based accelerometers and gyroscopes (body-worn)	Physical activities: walking, running, sitting; Postural transitions: sitting down, standing up, lying down	Strong focus on physical activities and postural changes. Underrepresented: complex ADLs, household tasks, cognitive activities, social interactions, manipulation tasks	I	B, C
UCI HAR Dataset [26]	The data collection protocol—30 volunteers aged 19 to 48 wearing Samsung Galaxy S II smartphones on their waists; 3-axis accelerometer and gyroscope signals sampled at 50 Hz during six ADLs (walking, walking upstairs, walking downstairs, sitting, standing, lying). It also describes sensor pre-processing (noise filtering, gravity separation) and feature extraction across sliding time windows, establishing the dataset as a benchmark for smartphone-based activity recognition.	3-axis accelerometer and gyroscope from smartphone (waist-worn, Samsung Galaxy S4)	6 basic activities: walking, walking upstairs, walking downstairs, sitting, standing, lying	Very limited activity variety (only 6 basic postural/ambulatory activities). Missing: manipulation tasks, household activities, social interactions, cognitive tasks, outdoor activities, complex ADLs	I	B
Skoda Mini Checkpoint [27]	This dataset captures technical gestures performed in industrial assembly line settings using accelerometers placed on both arms of a worker. It includes ten distinct manipulation gestures—such as opening a car bonnet or checking the boot—each repeated approximately 70 times over a three-hour recording session, sampled at around 98 Hz using 20 accelerometers mounted on the upper and lower arms.	20 accelerometers (upper and lower arms, bilateral placement)	10 industrial manipulation gestures: reaching, bending, picking, lifting, checking bonnet, checking boot, assembly related movements	Highly specialized for industrial/manufacturing contexts. Missing: general ADLs, whole-body activities, locomotion variety, household tasks, social interactions, lower body movements	I	C
Daphnet Gait Dataset [28]	This dataset focuses on gait analysis and the detection of freezing of gait (FoG) episodes in Parkinson’s disease patients. It contains accelerometer data recorded from sensors placed on the lower limbs and lower back of participants during walking tasks in a controlled environment. The dataset is widely used to develop and evaluate algorithms for early FoG detection and mobility monitoring in clinical and assistive settings.	3-axis accelerometers (lower limbs and lower back)	Gait analysis: normal walking, walking with freezing of gait (FoG) episodes, turns, walking tasks in Parkinson’s patients	Highly specialized for Parkinson’s disease gait patterns. Missing: other ADLs, upper body activities, general population data, healthy subject comparisons, non-gait activities, diverse mobility conditions	I	CA
SHL (Smartphone-Based Human Activity Recognition) [29]	The SHL dataset contains sensor data collected from smartphones placed at various body locations under realistic urban mobility conditions. It includes accelerometer, gyroscope, magnetometer, and GPS signals recorded during activities such as walking, cycling, riding public transport, and driving. The dataset is designed to support the development of activity recognition systems in real-world, dynamic environments.	Accelerometer, gyroscope, magnetometer, GPS, and additional sensors from smartphone (multiple body locations: hand, torso, bag, pocket)	Transportation modes: walking, running, cycling, bus, train, car, subway, standing still; urban mobility activities	Strong focus on transportation/mobility activities. Underrepresented: static indoor activities, household tasks, fine manipulation, social interactions, complex ADLs, non-transportation contexts, sedentary behaviors	I, E	B, SC
Kinect Activity Recognition Dataset [30]	This dataset uses Kinect depth cameras to capture human activities in indoor environments. It includes skeletal joint data for actions such as sitting down, standing up, walking, and picking up objects. The dataset is commonly used to train and evaluate activity recognition models that rely on 3D pose estimation and motion analysis.	Kinect depth camera (skeletal tracking, 3D joint positions, depth sensing)	Basic actions: sitting down, standing up, walking, waving, clapping, picking up objects, simple gestures	Limited to simple, isolated actions in controlled indoor settings. Missing: complex multi-step ADLs, fine manipulation details, outdoor activities, occlusion handling, multi-person interactions, naturalistic scenarios, sensor diversity beyond vision	V	B, C
IMU-LOC Dataset [31]	The IMU-LOC dataset is a multimodal resource that combines inertial sensor readings with location information for human activity recognition in indoor environments. It captures synchronized tri-axial accelerometer and gyroscope data from smartphone-mounted IMUs at approximately 64 Hz during six daily activities—walking, walking upstairs/downstairs, jogging, sitting, and standing—along with positional context provided by Raspberry Pi–based localization units. The dataset, comprising nearly 15,980 time-stamped observations, supports research in activity classification and context-aware health monitoring.	Smartphone-mounted IMUs (accelerometer and gyroscope at ~64 Hz), Raspberry Pi-based localization system	6 activities: walking, walking upstairs, walking downstairs, jogging, sitting, standing	Limited activity diversity (only 6 basic ambulatory/postural activities). Missing: complex ADLs, household tasks, manipulation activities, social interactions, lying down, outdoor activities, transitional movements, cognitive tasks	I, E	B, SC
ExtraSensory Dataset [32]	Collected in real-world conditions using participants’ own smartphones and smartwatches, the ExtraSensory dataset includes multi-minute sensor recordings from 60 users. It features accelerometer, gyroscope, magnetometer, GPS, audio, and phone-state data, and is annotated with self-reported labels spanning over 50 daily activities and contexts.	Accelerometer, gyroscope, magnetometer, GPS, audio, phone-state data from smartphones and smartwatches (participants’ own devices)	Multi-context activities: walking, running, sitting, standing, lying, driving, cycling, eating, cooking, watching TV, working, exercising; Context labels: at home, at work, outdoors, indoors, with people	Reliance on self-reported labels (potential annotation inconsistency). Activity labels may be coarse-grained. Missing: fine-grained manipulation tasks, detailed household activities, clinical populations, synchronized ground truth for complex activities	I, E, B	B, SC, C
TUM Kitchen Dataset [33]	The TUM Kitchen Dataset captures everyday manipulation activities in a realistic kitchen setting. It comprises multimodal data—including RGB video recordings from four overhead cameras (25 fps, 384 × 288 resolution), markerless full-body motion capture (51-DoF), RFID tag readings, and magnetic sensor readings—all synchronized and annotated for tasks such as table setting. It serves as a benchmark for motion segmentation and action recognition in complex, real-world environments.	RGB-D cameras (4 overhead), markerless full-body motion capture (31-DoF), RFID tag readers, magnetic sensors	Kitchen manipulation activities: table setting, reaching for objects, opening/closing drawers and cabinets, pouring, stirring, cutting, placing objects	Focus limited to kitchen environment only. Missing: other household rooms/contexts, outdoor activities, social interactions, non-manipulation ADLs (hygiene, dressing), ambulatory activities, diverse populations, long-duration tasks	V, E, MP	C, SC
USC-HAD (USC Human Activity Dataset) [34]	This dataset records indoor human activities using wearable inertial sensors placed on different body parts. It includes data from accelerometers and gyroscopes during defined daily actions—such as walking, sitting, and climbing stairs—captured from multiple participants. USC-HAD supports comparative evaluations of HAR algorithms in healthcare and general-purpose monitoring contexts.	Accelerometers and gyroscopes (wearable inertial sensors on body)	Basic physical activities: walking, running, jumping, climbing stairs (upstairs/downstairs), sitting, standing, elevator up/down, sleeping	Limited to basic ambulatory and postural activities. Missing: complex ADLs, household tasks, manipulation activities, social interactions, cognitive tasks, outdoor contexts, fine-grained movements, transitional activities, natural daily routines	I	B
MotionSense Dataset [35]	This dataset captures accelerometer and gyroscope data from an iPhone 6s placed in the front pants pocket of participants during six physical activities: walking, jogging, sitting, standing, walking upstairs, and walking downstairs. Data were collected at 50 Hz over 15 trials per subject, involving 24 individuals with diverse gender, age, height, and weight. MotionSense supports both human activity recognition and the inference of personal attributes from motion patterns.	Accelerometer and gyroscope from iPhone 6s (front trouser pocket, 50 Hz sampling rate)	6 basic activities: walking, jogging, sitting, standing, going upstairs, going downstairs	Very limited activity diversity (only 6 basic ambulatory/postural activities). Missing: complex ADLs, household tasks, manipulation activities, lying down, social interactions, outdoor contexts beyond walking, fine motor skills, transitional movements, upper body activities	I	B
UTD-MHAD [36]	The UTD-MHAD dataset comprises synchronized multimodal data—RGB videos, depth frames, 3D skeletal joint positions from a Kinect camera, and inertial readings from a wearable sensor—for 27 distinct human actions performed by 8 subjects. Each action is repeated multiple times (typically four), creating a comprehensive set of 861 action sequences. The dataset is particularly valuable for researching sensor fusion between depth and inertial modalities in indoor activity recognition tasks.	RGB camera, depth camera (Kinect), skeletal joint positions (Kinect), wearable inertial sensor (accelerometer, typically on wrist)	27 actions: swipe left/right, wave, clap, throw, arm cross, basketball shoot, draw X/circle, bowling, boxing, baseball swing, tennis swing/serve, arm curl, tennis serve, push, knock, catch, pickup/throw, jog, walk, sit-to-stand, stand-to-sit, lunge, squat	Focus on isolated, repetitive actions in controlled settings. Missing: naturalistic ADLs, complex multi-step tasks, household activities, social interactions, fine manipulation, continuous daily routines, outdoor contexts, cognitive activities	I, V	C
UCI Gas Sensor Array Dataset for HAR [37]	This dataset includes recordings from an array of 8 metal-oxide gas sensors, plus temperature and humidity sensors, deployed in a simulated indoor kitchen environment. The sensor data captures changes in air composition resulting from human activities—such as cooking, cleaning, or movement—making it a unique resource for non-intrusive activity recognition using ambient sensor signals.	8 metal-oxide gas sensors (VOC detection), temperature sensor, humidity sensor	Kitchen-related activities inferred from gas emissions: cooking, cleaning, presence/movement in kitchen environment	Highly specialized sensing modality (gas sensors). Limited to kitchen context only. Missing: direct activity labels, non-kitchen environments, fine-grained activity details, visual confirmation, wearable integration, other household rooms, outdoor activities, social contexts	E	SC
KU-HAR [38]	The dataset contains over 600,000 time-domain sensor records capturing 18 daily activities performed by 90 participants. Data were collected via built-in smartphone accelerometers and gyroscopes at 100 Hz, with devices positioned in a waist pouch. It includes 1945 raw activity samples and approximately 20,750 three-second subsamples, making it suitable for evaluating classification models in real-world HAR scenarios.	Built-in smartphone accelerometers and gyroscopes (waist pouch position)	18 daily activities: walking, standing, sitting, lying, stand-to-sit, sit-to-stand, sit-to-lie, lie-to-sit, stand-to-lie, lie-to-stand, walking upstairs, walking downstairs, running, jumping, phone call, texting, typing, writing	Good coverage of basic ADLs and postural transitions. Missing: complex household tasks (cooking, cleaning details), social interactions, cognitive activities, fine manipulation tasks, outdoor activities beyond walking/running, context-rich scenarios	I	B, C
Berkeley MHAD (Multimodal Human Action Database) [39]	This richly multimodal dataset comprises synchronized data from 12 RGB cameras, 2 Kinect depth sensors, 6 wearable accelerometers, an optical motion-capture system, and 4 microphones. It includes 659 sequences of 11 distinct actions—each performed five times—by 12 subjects in a controlled indoor environment. It serves as a standard benchmark for developing and testing algorithms that utilize both vision-based and inertial signals for action recognition.	12 RGB cameras, 8 RGB-D sensors (depth), 6 wearable accelerometers, optical motion-capture system (marker-based), 4 microphones	11 actions: jumping in place, jumping jacks, bending, punching, waving (two hands/one hand), clapping hands, throwing ball, sit-to-stand, stand-to-sit	Limited to 11 simple, isolated actions in highly controlled lab setting. Missing: complex ADLs, household tasks, naturalistic behaviors, fine manipulation, cognitive activities, social interactions, outdoor contexts, continuous daily routines, elderly/clinical populations	I, V, B	C
DLR IMU Activity Dataset [40]	Collected by the Institute of Communications and Navigation at the German Aerospace Center (DLR), this dataset comprises motion data from a single inertial measurement unit (IMU) worn on the belt of 16 participants (mixed gender, ages 23–50). The IMU sampled tri-axial acceleration and rotation rate at 100 Hz across seven activities including walking, running, standing, sitting, lying, jumping, and falling. Approximately 4.5 h of labelled recordings support research in real-time activity and fall detection using minimal, single-sensor setups.	Single IMU (tri-axial accelerometer and gyroscope, 102.4 Hz sampling rate, body-worn)	Activities: walking, running, standing, sitting, lying, jumping, falling (falls). Focus on routine activities and fall events	Limited activity variety focused mainly on ambulatory activities and falls. Missing: complex ADLs, household tasks, manipulation activities, social interactions, cognitive tasks, transitional movements beyond basic postures, upper body activities, fine motor skills	I	B, C

Conventions: Sensor Type(s): Environmental Sensors (E), Motion and Proximity Sensors (MP), Inertial Sensors (I), Biometrics Sensors(B),Vision Sensors (V); Activity Type(s): Basic (B), Social/contextual (SC), Complex (C), Critical or Anomalous Activities (CA).

**Table 4:** Types of Transfer Learning Algorithms in HAR.

Transfer Learning Type	Description	Representative Methods
Feature-Based	Transfers shared representations between source and target using domain-invariant feature learning.	DANN, MMD, CORAL [45,46]
Instance-Based	Selects or reweights instances from the source domain to match the target distribution.	TrAdaBoost, KMM [47]
Parameter-Based	Fine-tunes model parameters from a pretrained network using target domain data.	CNN/RNN fine-tuning, TransferCNN [48]
Relational Transfer	Transfers temporal, structural, or semantic relationships among activities or features across domains.	Graph-based transfer, relational modeling [49]

**Table 5:** Types of Reinforcement Learning Algorithms in HAR.

Dataset	Description
Q-Learning [51]	Q-learning is one of the most widely used reinforcement learning algorithms. In HAR, it can be applied to optimize decision-making processes such as dynamically adjusting sensor configurations or selecting features that maximize recognition accuracy. Q-learning operates by updating a Q-table that stores the expected future rewards for each action-state pair, enabling the system to learn optimal actions over time.
Deep Q-Networks (DQN) [52]	Deep Q-Networks (DQNs) extend traditional Q-learning by incorporating deep neural networks to approximate Q-values in high-dimensional state spaces. This approach is particularly effective in HAR scenarios, where the state space is large due to the diversity of activities and sensor inputs. DQNs enable the system to learn complex activity representations without relying on explicit feature engineering.
Policy Gradient Methods [53]	These methods optimize a policy directly by estimating the gradient of the expected reward with respect to the policy parameters. In HAR, policy gradient algorithms can be employed to optimize continuous control tasks, such as adjusting sensor thresholds or dynamically selecting appropriate sensor modalities in real time. Algorithms such as REINFORCE and Proximal Policy Optimization (PPO) are frequently used in these applications.
Multi-Agent Reinforcement Learning (MARL) [54]	In environments involving multiple agents (e.g., sensors or devices), Multi-Agent Reinforcement Learning (MARL) enables each agent to learn its own policy while accounting for the actions of other agents. This approach is particularly valuable in smart home systems or wearable sensor networks, where multiple devices must collaborate to accurately recognize human activities.
Inverse Reinforcement Learning (IRL) [55]	Unlike traditional reinforcement learning, which learns from explicit reward signals, Inverse Reinforcement Learning (IRL) infers the underlying reward function from expert demonstrations. In HAR, IRL can be applied to learn from human behavior, enabling the system to recognize complex activities that are difficult to formalize through predefined rules.

**Table 6:** Key advancements in the field between 2010–2016.

Ref.	Year	Author	Technical Evaluation
[63]	2010	Sim et al.	The system shows strong integration of multiple sensor modalities and employs an MDP with Q-learning to improve the recognition of erroneous plans in ADLs, achieving a 26.2% improvement in F-measure. Its main limitations lie in scalability, limited validation in real-world settings, and sensitivity to initial conditions. Technically, it exemplifies the use of Reinforcement Learning in HAR, where Q-learning filters sensor noise and supports decision-making. Although Transfer Learning is not applied, the system’s modular design suggests potential for adaptation to new environments with minimal manual intervention.
[64]	2013	Pietquin	Proposes the use of Inverse Reinforcement Learning (IRL) to model interactive systems, particularly in spoken dialogue management. The paper explores how IRL can simulate user behavior, cluster interactions, and achieve human-machine co-adaptation by learning the reward function directly from expert demonstrations. Technically, it highlights IRL’s strength in removing the need for manually designed rewards and representing user policies compactly via feature expectations. It also allows comparing and generalizing behaviors across users and systems, supporting transferability. However, IRL remains computationally intensive and requires subsequent policy optimization after learning the reward. The method is promising for emulating human-like interaction but lacks quantitative validation in HAR-specific settings.
[65]	2014	Prins	The authors propose an actor-critic approach for brain-machine interfaces (BMI), introducing a confidence metric that regulates system updates based on the reliability of the critic’s feedback. This technique significantly improves performance under noisy and uncertain conditions, achieving up to 70% accuracy even with unreliable critics. Its modular architecture and ability to operate with unstructured biological signals position it as a reference point for the development of current adaptive systems based on reinforcement learning.
[66]	2016	Saeedi	A set of transfer learning algorithms is proposed to enable the autonomous reconfiguration of wearable systems in human activity recognition tasks. These methods allow pre-trained models to be adapted to new users or devices without requiring retraining, using techniques such as motif-based mapping and similarity metrics. Experimental results show accuracy improvements of up to 13% in cross-subject and cross-sensor transfer scenarios. This approach represents a significant advancement in transfer learning, particularly for mobile and heterogeneous environments.
[67]	2016	Wei	A novel Co-Regularized Heterogeneous Transfer Learning (CoHTL) model is proposed to address the sparsity of labeled sensor data in HAR by transferring knowledge from social media. By projecting both physical sensor data and social messages into a shared latent semantic space, the model aligns heterogeneous domains despite differences in feature spaces and label representations. Through co-regularization and matrix factorization, CoHTL preserves intra-domain structures and semantic inter-domain similarities. Technically, this method outperforms state-of-the-art baselines such as HeMap and DAMA, achieving up to 25% improvement in classification accuracy under limited labeled data. Its ability to enrich physical sensor features using social signals represents a significant advance in transfer learning for ubiquitous computing applications, particularly in contexts where sensor labeling is expensive or impractical.

**Table 7:** Development Phase between 2017–2020.

Ref.	Year	Author	Technical Evaluation
[4]	2017	Wang	Wang makes a significant contribution to the field of human activity recognition by developing a kernel fusion-based Extreme Learning Machine (ELM) model for cross-location activity recognition. This approach addresses a key challenge in the field: the variability of locations where data is collected. The kernel fusion technique enables the integration of diverse information sources, thereby improving the model’s accuracy in recognizing activities across different environments and enhancing its generalization capability.
[68]	2017	Khan	They propose TransAct, an activity recognition model based on transfer learning and clustering. It employs the Instance-Based Transfer Boost algorithm combined with anomaly detection and k-means clustering. The model is evaluated using the HAR, MHealth, and DailyAndSports datasets. It achieves an average accuracy of 81.55%, outperforming traditional methods such as Random Forest (68.20%) and Decision Tree (63.81%). Its main strength lies in its ability to recognize new activities in environments with limited labeled data.
[2]	2018	Ramasamy	Ramasamy provides a comprehensive review of recent trends in the application of machine learning techniques for HAR. The work offers an overview of the most advanced and emerging methods in the field, with a particular focus on deep learning techniques, recurrent neural networks, and transfer learning approaches. In addition, the article addresses current challenges and outlines future research directions in HAR, offering valuable guidance for researchers aiming to enhance the accuracy and effectiveness of activity recognition systems.
[5]	2018	Khan	The authors introduce HDCNN, a transductive adaptation model based on convolutional neural networks (CNNs), designed to scale human activity recognition (HAR) across different domains without requiring labeled data in the target domain. It operates under the assumption that the relative distribution of CNN weights remains invariant when the underlying activities are consistent. The model achieves high accuracy even in the absence of target labels, and with minimal labeled data, it significantly outperforms both shallow and deep baseline classifiers.
[6]	2019	Seyfioglu	Seyfioglu makes an innovative contribution by applying transfer learning in deep neural networks (DNNs) for motion classification using diverse micro-Doppler data. The study demonstrates that transferring learned features across different micro-Doppler scenarios enhances the accuracy of motion classification in radar systems. This approach is particularly valuable for aerospace and electronic systems applications, where precise motion classification is essential for the reliable operation of detection and tracking systems.
[69]	2019	Ozcan	The authors propose an architecture based on transfer learning (using a pre-trained AlexNet model) and hyperparameter optimization through heuristic algorithms, including Artificial Bee Colony (ABC), Genetic Algorithms, and Particle Swarm Optimization (PSO). Experiments were conducted on the Sign Language Digits and Thomas Moeslund Gesture Recognition datasets, with each experiment repeated 30 times. The ABC method achieved an average accuracy of 98.40% on the first dataset (compared to the previous state-of-the-art at 94.2%) and 98.09% on the second (compared to 94.33%). The results demonstrate clear superiority over previous approaches using both deep and traditional techniques.
[1]	2020	Zhou	The authors introduce a semi-supervised framework powered by Deep Q-Networks (DQN) for automatic labeling, combining data from multiple body-worn and contextual sensors. They apply a multimodal fusion technique along with an LSTM network to classify fine-grained patterns in sequential data. The model is evaluated using real-world datasets, showing substantial improvements in both accuracy and efficiency in environments with limited labeled data.
[70]	2020	Wilson	Wilson contributes to the field of human activity recognition by proposing a multi-source deep domain adaptation method with weak supervision for time-series sensor data. This approach enables knowledge transfer from multiple source domains to a target domain with limited data and partial labeling, thereby improving activity classification accuracy. The method is particularly useful in applications where labeled data is scarce or costly to obtain, enhancing performance in real-world scenarios.

**Table 8:** Strengthening Phase between 2021–today.

Ref.	Year	Author	Technical Evaluation
[10]	2021	Soleimani	Soleimani makes a significant contribution to the field of HAR by implementing cross-subject transfer learning through Generative Adversarial Networks (GANs). This methodology tackles one of the central challenges in HAR: the inter-subject variability that often hampers model accuracy when applied to unseen users. By leveraging GANs, the authors introduce a technique that enables the transfer of learned knowledge from one group of subjects to another, thereby enhancing the adaptability of recognition systems to diverse individuals without the need for extensive labeled datasets.
[71]	2021	Khan	Khan and Ghani provide a comprehensive survey of deep learning models applied to HAR. The paper evaluates various architectures, including CNNs, RNNs, hybrid models, and GAN-based approaches. It compares performance across standard datasets (e.g., WISDM, UTD-MHAD, OPPORTUNITY) using common metrics such as accuracy and F1-score. The authors highlight major challenges like inter-subject variability, data labeling requirements, and generalization issues. They also suggest future directions focusing on model robustness, transfer learning, and real-time implementation.
[45]	2022	Li	Li makes a notable contribution to the field of Human Activity Recognition by applying semi-supervised learning techniques to micro-Doppler signatures obtained from radar. This approach enables the detection and classification of human activities using minimal labeled data, while effectively leveraging unlabeled information to improve model performance. The proposed methodology is particularly valuable in scenarios where obtaining large volumes of labeled data is costly or impractical, thereby enhancing the applicability and robustness of recognition systems in real-world environments.
[72]	2022	Ariza	They review the historical evolution and current approaches in data analysis for HAR, including supervised, unsupervised, ensemble, deep, reinforcement, transfer, and metaheuristic learning methods. The study examines recent experimental metrics and identifies promising directions—particularly reinforcement and transfer learning—applicable to Ambient Assisted Living environments, highlighting key challenges and emerging trends.
[46]	2023	Ray	Ray conducts a comprehensive decade-long analysis of vision-based Human Activity Recognition, enhanced through transfer learning. This study highlights how transfer learning techniques have boosted the accuracy and effectiveness of activity recognition systems, enabling the adaptation of pre-trained models to new environments with limited data. The article not only examines technological developments in this field but also provides a thorough overview of emerging trends and future challenges in Human Activity Recognition using vision-based approaches.
[73]	2023	Sahoo	The authors develop a HAR model based on wearable sensors by converting accelerometer and gyroscope signals into spectrogram images. Deep features are extracted using pre-trained CNNs, and a wrapper-based method (BBA) is applied for feature selection. The approach yields substantial improvements in accuracy (+21%, +20%, +6%) using only 52–60% of the original features, significantly reducing training time and enhancing overall performance on HARTH, KU-HAR, and HuGaDB datasets.
[74]	2024	Hassan	They propose a dynamic HAR method that combines pre-trained CNN-based feature extraction (using MobileNetV2) with a deep bidirectional LSTM (Deep BiLSTM) classifier. Thanks to transfer-learning-based features and iterative fine-tuning, the model achieves high accuracy on three video benchmarks: UCF11 (99.20%), UCF Sport (93.3%), and JHMDB (76.30%).
[75]	2024	Kaseris	This survey delivers an extensive overview of deep learning and classical machine learning techniques used in HAR, covering multiple input modalities including wearable sensors (accelerometer/gyroscope), video, and audio. It introduces a novel method using large language models (LLMs) to filter and rank relevant literature, offering a clear taxonomy of current methods. The paper is particularly valuable for organizing the HAR landscape, discussing historical evolution, modality fusion, datasets, methodological trends, and future directions.
[76]	2025	Thukral	The authors introduce Cross-Domain HAR, a novel few-shot transfer learning framework following a teacher–student self-training paradigm. It bridges gaps across source and target domains (sensor placement, activity types), using self-supervision, consistency regularization, and data augmentation. Evaluated across six IMU datasets, the method delivers substantial improvements (~20% gain with only 2–5 labeled samples per class) compared to strong baselines, demonstrating robust few-shot adaptation in realistic HAR scenarios.
[77]	2025	Lamani	The authors introduce HARNet-SVM, a novel lightweight residual 3D CNN (HARNet) built on directed acyclic graphs to jointly learn spatial and motion representations from raw video. Extracted latent features from HARNet’s fully connected layer are fed into an SVM classifier, yielding efficient action recognition. The method demonstrates significant performance improvements: +2.75% on UCF101, +10.94% on HMDB51, and +0.18% on KTH, compared to current state-of-the-art models, while reducing computational complexity.

**Table 9:** Leading Countries in Research Publications.

Country	Production		Citation		Q1	Q2	Q3	Q4
China	51	17.65%	451	15.34%	15	2	3	1
Usa	41	14.19%	699	23.78%	7	2	1	1
India	29	10.03%	127	4.32%	3	4	1	2
United Kingdom	17	5.88%	113	3.84%	3	2	1	0
Korea	16	5.54%	132	4.49%	4	4	0	0
Japan	14	4.84%	305	10.37%	2	1	1	0
Germany	11	3.81%	92	3.13%	2	2	0	0
Canada	8	2.77%	4	0.14%	0	1	0	0
Iran	8	2.77%	66	2.24%	1	2	3	0
Singapore	8	2.77%	229	7.79%	2	0	0	0

**Table 10:** Top Ten Journals.

Journal	Wos	Scopus	Wos and Scopus	Impact Factor (SJR)	Impact Factor (JCR)	H Index	Quartile (SJR)	Quartile (JCR)	ICORE Rank (If Conference)
Sensors	18	14	14	0.76		219	Q1	Q2	--
Lecture Notes In Computer Science (Including Subseries Lecture Notes In Artificial Intelligence And Lecture Notes In Bioinformatics)	0	14	14	0.32		446	Q2	--	__
IEEE Internet of Things Journal	7	7	7	3.75		149	Q1	Q1	
ACM International Conference Proceeding Series	0	7	7	0.21		137	-	--	B
Neural Computing And Applications	0	6	6	1.17		111	Q1	Q2	--
Computers, Materials And Continua	0	5	5	0.53		51	Q2	Q3	--
Expert Systems With Applications	4	3	3	1.87		249	Q1	Q1	--
IEEE Access	5	5	3	0.93		204	Q1	Q2	--
Proceedings—International Symposium On Wearable Computers, Iswc	0	5	5	0		57	-	--	--
Communications In Computer And Information Science	0	4	4	0.19		62	Q4	--	--

**Table 11:** Production By Author.

No	Researcher	Total Articles *	H-Index (Scopus)	Affiliation
1	Chen Y	9	25	National Taipei University, Taipei, Taiwan
2	Khan M	8	112	Kennesaw State University, Kennesaw, United States
3	Roy N	8	20	University Of Maryland, Baltimore County (Umbc), Baltimore, United States
4	Wang J	8	21	Microsoft Research, Redmond, United States
5	Abootalebi V	6	12	Yazd University, Yazd, Iran
6	Kim Y	6	3	Inha University, Incheon, South Korea
7	Li J	6	1	Harbin Institute Of Technology, Harbin, China
8	Li X	6	7	University Of Technology Sydney, Sydney, Australia
9	Zebhi S	6	3	Yazd University, Yazd, Iran
10	Almodarresi S	5	6	Yazd University, Yazd, Iran

## Data Availability

The original contributions presented in this study are included in the article. Further inquiries can be directed to the corresponding author.

## Supplementary Materials

The following supporting information can be downloaded at: https://www.mdpi.com/article/10.3390/s26123751/s1.
