# Supplementary material for: Transfer and Reinforcement Learning as Support Paradigms for Human Activity Recognition in Indoor Environments: A Comprehensive Analysis of Trends, Impact and Future Directions
Source: Sensors (Basel). 2026 Jun 12;26(12):3751. doi: 10.3390/s26123751 (PMC13306968; doi:10.3390/s26123751)
Supplement: Supplementary file 1 [file sensors-26-03751-s001.zip › sensors-3714131-supplementary.pdf]

PRISMA 2020 Checklist

Table S1. PRISMA 2020 Checklist

Section and Topic	Item #	Checklist item	Location where item is reported
**TITLE Transfer and Reinforcement Learning as Support Paradigms for Human Activity Recognition in Indoor Environments: A Comprehensive Analysis of Trends, Impact and Future Directions**			Title page / manuscript header: the article type is identified as “Systematic Review”.
Title	1	Identify the report as a systematic review.	Title page / manuscript header: the article type is identified as “Systematic Review”.
**ABSTRACT**			
Abstract	2	See the PRISMA 2020 for Abstracts checklist.	Abstract, p. 1. See also the completed PRISMA 2020 for Abstracts checklist.
**INTRODUCTION**			
Rationale	3	Describe the rationale for the review in the context of existing knowledge.	Section 1. Introduction, pp. 2–3: rationale for HAR, healthcare relevance, research dispersion, and need for a systematic/scientometric perspective.
Objectives	4	Provide an explicit statement of the objective(s) or question(s) the review addresses.	Section 1. Introduction, p. 2: objective to analyze key trends in HAR with emphasis on transfer learning and reinforcement learning.
**METHODS**			
Eligibility criteria	5	Specify the inclusion and exclusion criteria for the review and how studies were grouped for the syntheses.	Section 3. Methods, p. 16: exclusion criteria are described; documents without original research/empirical data, non-English publications, and studies not directly related to HAR were excluded. Grouping for synthesis is described through scientometric analysis and Tree of Science categories in Sections 3.1–3.2.
Information sources	6	Specify all databases, registers, websites, organisations, reference lists and other sources searched or consulted to identify studies. Specify the date when each source was last searched or consulted.	Section 3. Methods, p. 16: Web of Science and Scopus databases are specified; search period from 2010 to the present. The exact last search date is not explicitly reported in the manuscript.
Search strategy	7	Present the full search strategies for all databases, registers and websites, including any filters and limits used.	Section 3. Methods, p. 16: Boolean query reported as (“Transfer Learning” OR “Reinforcement Learning”) AND (“HAR” OR “Human Activity Recognition” OR “ADL” OR “Activity Daily Living”), limited to title, abstract, and keywords in WoS and Scopus, 2010–present.
Selection process	8	Specify the methods used to decide whether a study met the inclusion criteria of the review, including how many reviewers screened each record and each report retrieved, whether they worked independently, and if applicable, details of automation tools used in the process.	Section 3. Methods, pp. 16–17 and Figure 1: records from Scopus and WoS were merged, duplicates removed, and records screened using the PRISMA-like flow. Number of reviewers and independent screening procedures are not explicitly reported.
Data collection process	9	Specify the methods used to collect data from reports, including how many reviewers collected data from each report, whether they worked independently, any processes for obtaining or confirming data from study investigators, and if applicable, details of automation tools used in the process.	Section 3. Methods, pp. 16–17: data integration and cleaning were performed using Bibliometrix, VOSviewer, RStudio, and Core of Science scripts. Number of reviewers and independent extraction procedures are not explicitly reported.
Data items	10a	List and define all outcomes for which data were sought. Specify whether all results that were compatible with each outcome domain in each study were sought (e.g. for all measures, time points, analyses), and if not, the methods used to decide which results to collect.	Sections 3.1, 4, 5 and 6: outcomes/indicators sought included annual production, citation impact, countries, journals, authors, co-authorship/citation networks, and Tree of Science categories (roots, trunk, branches/leaves).
	10b	List and define all other variables for which data were sought (e.g. participant and intervention characteristics, funding sources). Describe any assumptions made about any missing or unclear information.	Sections 3.1, 4, 5 and 6; Tables 6–10 and Figures 2–6: variables included author, year, reference, country, journal, citation indicators, technical contribution, datasets/algorithmic approaches, and thematic classification. Assumptions for missing/unclear data are not explicitly reported.
Study risk of bias assessment	11	Specify the methods used to assess risk of bias in the included studies, including details of the tool(s) used, how many reviewers assessed each study and whether they worked independently, and if applicable, details of automation tools used in the process.	Not reported / not applicable to this bibliometric-scientometric review. No formal individual-study risk-of-bias tool is described.
Effect measures	12	Specify for each outcome the effect measure(s) (e.g. risk ratio, mean difference) used in the synthesis or presentation of results.	Section 3.1 and Results sections: bibliometric/scientometric indicators are used instead of clinical effect measures. No meta-analysis or effect-size synthesis was performed.
Synthesis methods	13a	Describe the processes used to decide which studies were eligible for each synthesis (e.g. tabulating the study intervention characteristics and comparing against the planned groups for each synthesis (item #5)).	Section 3. Methods, pp. 16–17 and Figure 1: eligibility for synthesis was determined after database search, duplicate removal, exclusion screening, and organization into scientometric analysis and Tree of Science analysis.

Section and Topic	Item #	Checklist item	Location where item is reported
	13b	Describe any methods required to prepare the data for presentation or synthesis, such as handling of missing summary statistics, or data conversions.	Section 3. Methods, pp. 16–17: data were merged, cleaned, and deduplicated using Bibliometrix/R tools; 403 duplicates were removed before analysis.
	13c	Describe any methods used to tabulate or visually display results of individual studies and syntheses.	Sections 2–6: results are presented through descriptive tables and figures, including dataset tables, PRISMA flow diagram, scientific production/citation figures, country/journal/author analyses, and Tree of Science representation.
	13d	Describe any methods used to synthesize results and provide a rationale for the choice(s). If meta-analysis was performed, describe the model(s), method(s) to identify the presence and extent of statistical heterogeneity, and software package(s) used.	Sections 3.1 and 3.2: synthesis was conducted using scientometric analysis and the Tree of Science metaphor. Tools reported include Bibliometrix, VOSviewer, RStudio, tosr, and Core of Science scripts. No meta-analysis was performed.
	13e	Describe any methods used to explore possible causes of heterogeneity among study results (e.g. subgroup analysis, meta-regression).	Not applicable / not reported. The manuscript does not conduct subgroup analysis, meta-regression, or statistical heterogeneity exploration.
	13f	Describe any sensitivity analyses conducted to assess robustness of the synthesized results.	Not conducted / not reported. The manuscript does not describe sensitivity analyses.
Reporting bias assessment	14	Describe any methods used to assess risk of bias due to missing results in a synthesis (arising from reporting biases).	Not assessed / not reported. The manuscript does not describe a formal assessment of reporting bias or missing results.
Certainty assessment	15	Describe any methods used to assess certainty (or confidence) in the body of evidence for an outcome.	Not assessed / not reported. The manuscript does not describe a formal certainty-of-evidence assessment such as GRADE.
**RESULTS**			
Study selection	16a	Describe the results of the search and selection process, from the number of records identified in the search to the number of studies included in the review, ideally using a flow diagram.	Section 3. Methods, p. 16 and Figure 1, p. 17: search and selection process reported from 640 Scopus records and 165 WoS records, 805 preliminary records, duplicate removal, and final corpus for analysis.
	16b	Cite studies that might appear to meet the inclusion criteria, but which were excluded, and explain why they were excluded.	Section 3. Methods, p. 16: exclusion categories are reported, but individual excluded studies that might appear eligible are not cited one by one.
Study characteristics	17	Cite each included study and present its characteristics.	Tables 3, 6, 7, 8, 9 and related Results sections: included datasets and studies are cited and summarized with characteristics, technical evaluation, author, year, and reference.
Risk of bias in studies	18	Present assessments of risk of bias for each included study.	Not reported / not applicable. No individual-study risk-of-bias assessment is presented.
Results of individual studies	19	For all outcomes, present, for each study: (a) summary statistics for each group (where appropriate) and (b) an effect estimate and its precision (e.g. confidence/credible interval), ideally using structured tables or plots.	Tables 6–9 and Sections 4–5: individual studies are summarized narratively and technically. Effect estimates with confidence/credible intervals are not applicable because no meta-analysis was conducted.
Results of syntheses	20a	For each synthesis, briefly summarise the characteristics and risk of bias among contributing studies.	Sections 4–6 and Tables 6–9: characteristics of studies are summarized by historical phase and Tree of Science category. Risk of bias among contributing studies is not assessed.
	20b	Present results of all statistical syntheses conducted. If meta-analysis was done, present for each the summary estimate and its precision (e.g. confidence/credible interval) and measures of statistical heterogeneity. If comparing groups, describe the direction of the effect.	Sections 4–6 and Figures 2–6: bibliometric and scientometric syntheses are presented. No statistical meta-analysis, summary effect estimate, confidence interval, or heterogeneity statistic was conducted.
	20c	Present results of all investigations of possible causes of heterogeneity among study results.	Not applicable / not reported. No statistical investigation of heterogeneity was conducted.
	20d	Present results of all sensitivity analyses conducted to assess the robustness of the synthesized results.	Not applicable / not reported. No sensitivity analyses were conducted.
Reporting biases	21	Present assessments of risk of bias due to missing results (arising from reporting biases) for each synthesis assessed.	Not assessed / not reported. No formal reporting-bias assessment is presented.
Certainty of evidence	22	Present assessments of certainty (or confidence) in the body of evidence for each outcome assessed.	Not assessed / not reported. No certainty-of-evidence assessment is presented.
**DISCUSSION**			
Discussion	23a	Provide a general interpretation of the results in the context of other evidence.	Section 6. Discussion: Frontier of Knowledge in the Line of Research, pp. 42–49, interprets results using the Tree of Science framework and links them to the broader HAR literature.
	23b	Discuss any limitations of the evidence included in the review.	Section 3.1, pp. 17–18: limitations of the evidence/search are discussed, including keyword dependence, database coverage, terminology variation, and possible omission of emerging literature.

PRISMA

PRISMA 2020 Checklist

Section and Topic	Item #	Checklist item	Location where item is reported
	23c	Discuss any limitations of the review processes used.	Section 3.1, pp. 17–18: limitations of the review process are discussed, including reliance on WoS/Scopus and controlled search terms.
	23d	Discuss implications of the results for practice, policy, and future research.	Sections 6.3–6.6 and 7. Conclusions: implications for personalized healthcare, elderly care, real-time HAR, wearable sensors, domain adaptation, and future research are discussed.
**OTHER INFORMATION**			
Registration and protocol	24a	Provide registration information for the review, including register name and registration number, or state that the review was not registered.	Not registered / not reported. The manuscript does not provide a review register name or registration number.
	24b	Indicate where the review protocol can be accessed, or state that a protocol was not prepared.	Not prepared / not reported. The manuscript does not provide access to a review protocol.
	24c	Describe and explain any amendments to information provided at registration or in the protocol.	Not applicable. No registered protocol is reported; therefore, no amendments are described.
Support	25	Describe sources of financial or non-financial support for the review, and the role of the funders or sponsors in the review.	Funding statement: Grant PID2021-127275OB-I00 funded by MICIU/AEI/10.13039/501100011033 and by ERDF “A way of making Europe”.
Competing interests	26	Declare any competing interests of review authors.	Conflicts of Interest statement: the authors declare no conflicts of interest.
Availability of data, code and other materials	27	Report which of the following are publicly available and where they can be found: template data collection forms; data extracted from included studies; data used for all analyses; analytic code; any other materials used in the review.	Data Availability Statement: original contributions are included in the article; further inquiries can be directed to the corresponding author. Analytic code/templates are not reported as publicly available.

From: Page MJ, McKenzie JE, Bossuyt PM, Boutron I, Hoffmann TC, Mulrow CD, et al. The PRISMA 2020 statement: an updated guideline for reporting systematic reviews. BMJ 2021;372:n71. doi: 10.1136/bmj.n71. This work is licensed under CC BY 4.0. To view a copy of this license, visit https://creativecommons.org/licenses/by/4.0/
